# Supplementary figures and images for: CMTM3 regulates neutrophil activation and aggravates sepsis through TLR4 signaling
Source: EMBO Rep. 2024 Oct 25;25(12):5456–77. doi: 10.1038/s44319-024-00291-7 (PMC11624275; doi:10.1038/s44319-024-00291-7)

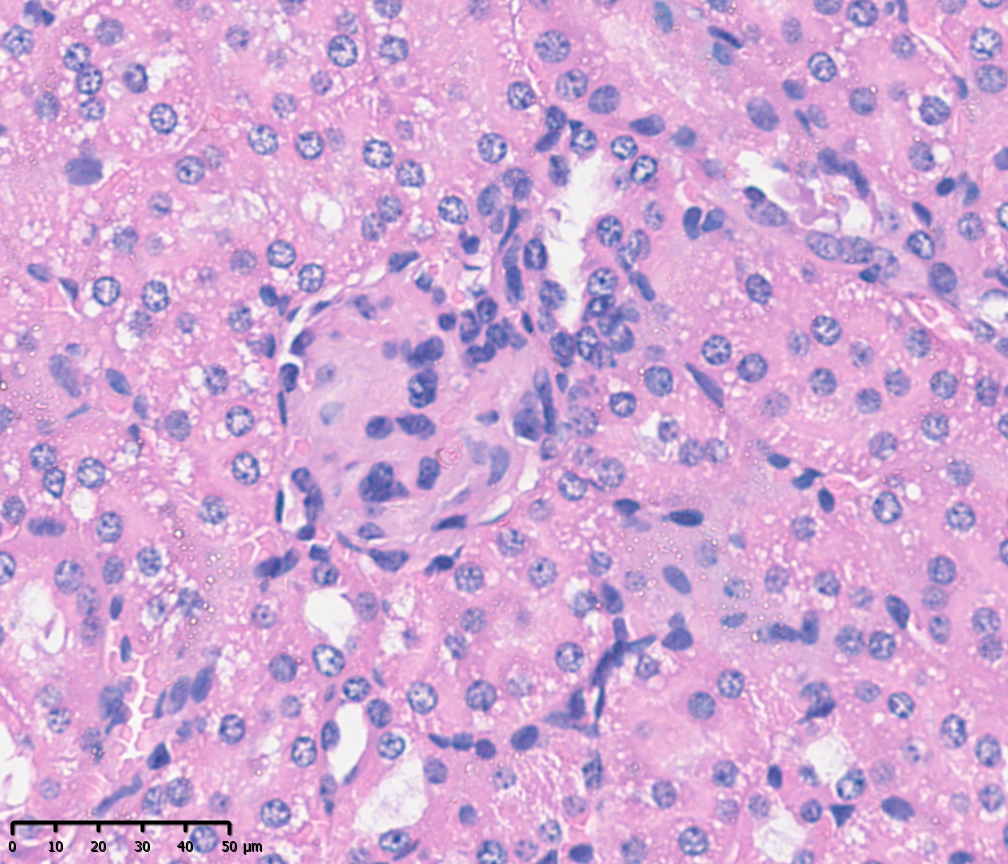

Supplement: Supplementary file 3 — Source data Fig. 2 [file 44319_2024_291_MOESM3_ESM.zip › Figure2G/KO_CLP_KIDNEY.tif]

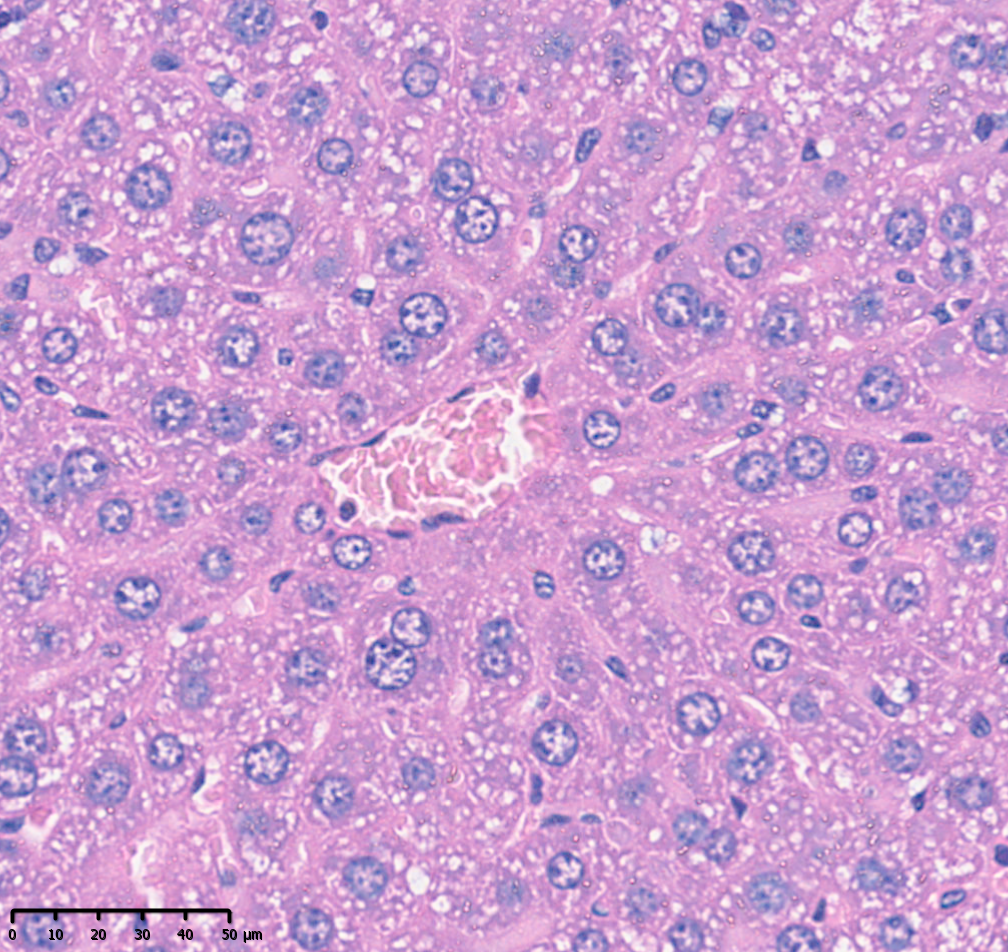

Supplement: Supplementary file 3 — Source data Fig. 2 [file 44319_2024_291_MOESM3_ESM.zip › Figure2G/KO_CLP_LIVER.tif]

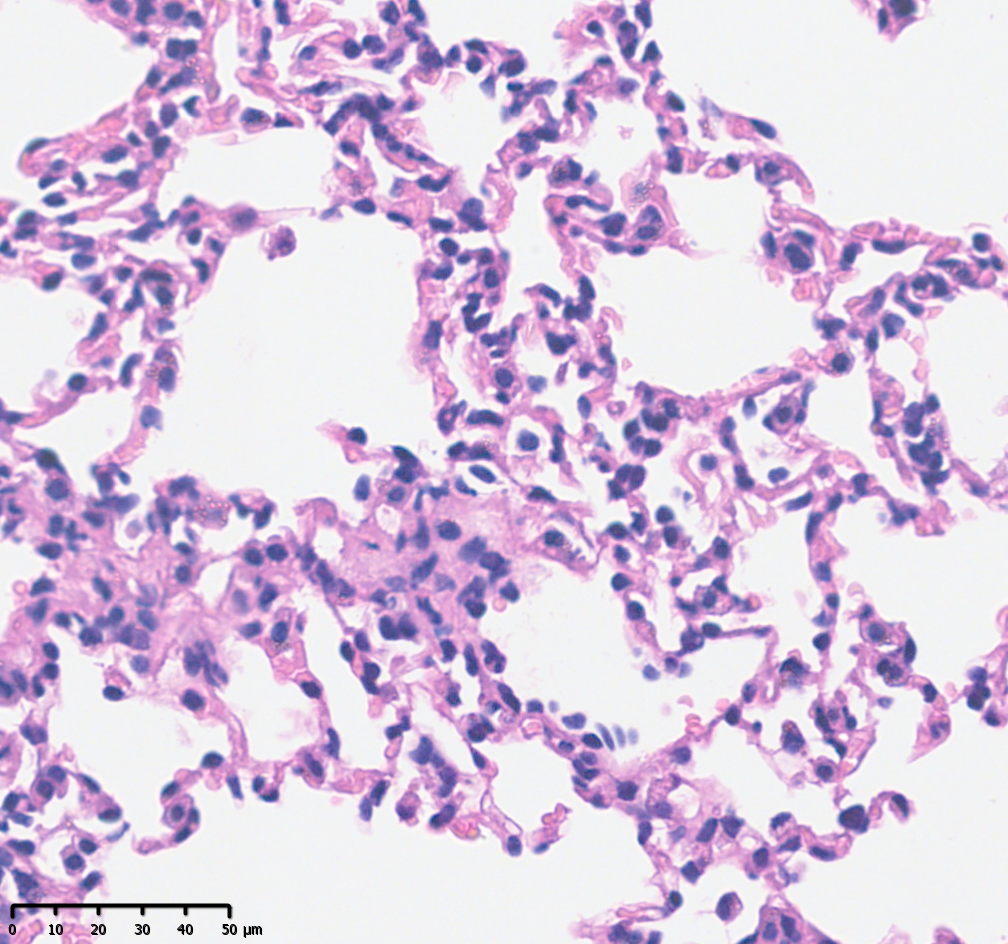

Supplement: Supplementary file 3 — Source data Fig. 2 [file 44319_2024_291_MOESM3_ESM.zip › Figure2G/KO_CLP_LUNG.tif]

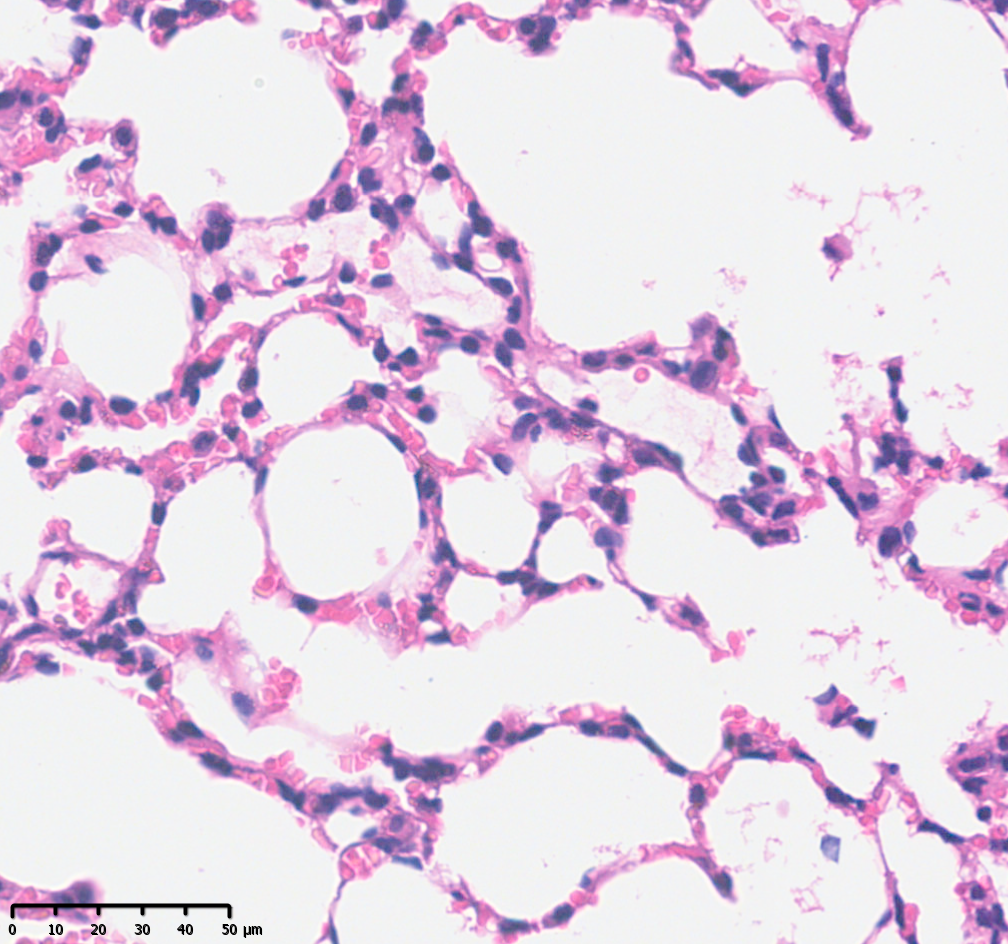

Supplement: Supplementary file 3 — Source data Fig. 2 [file 44319_2024_291_MOESM3_ESM.zip › Figure2G/KO_SHAM-LUNG.tif]

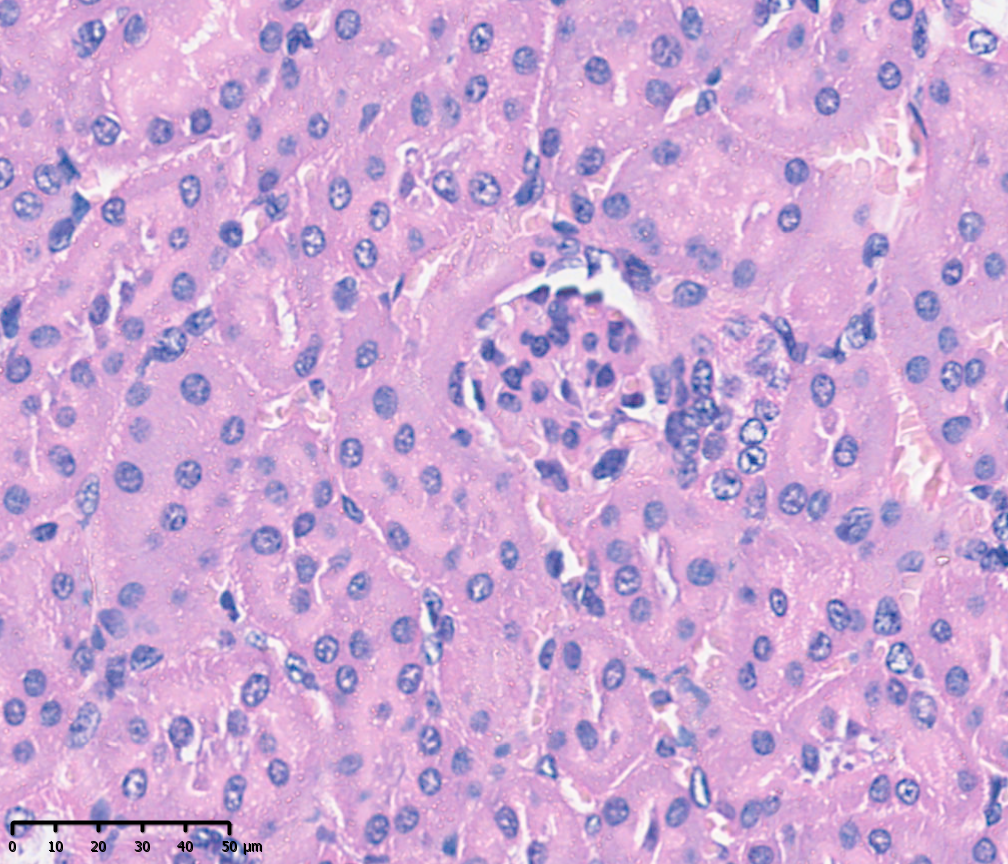

Supplement: Supplementary file 3 — Source data Fig. 2 [file 44319_2024_291_MOESM3_ESM.zip › Figure2G/KO_SHAM_KIDNEY.tif]

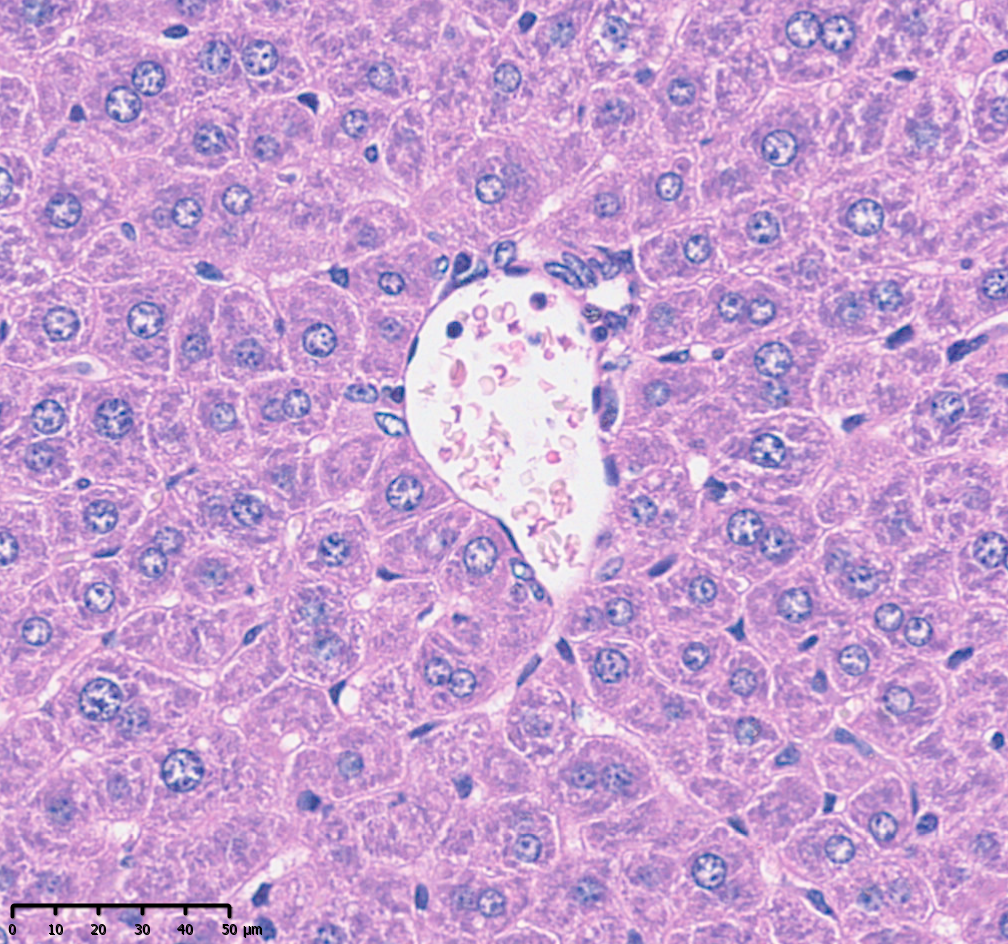

Supplement: Supplementary file 3 — Source data Fig. 2 [file 44319_2024_291_MOESM3_ESM.zip › Figure2G/KO_SHAM_LIVER.tif]

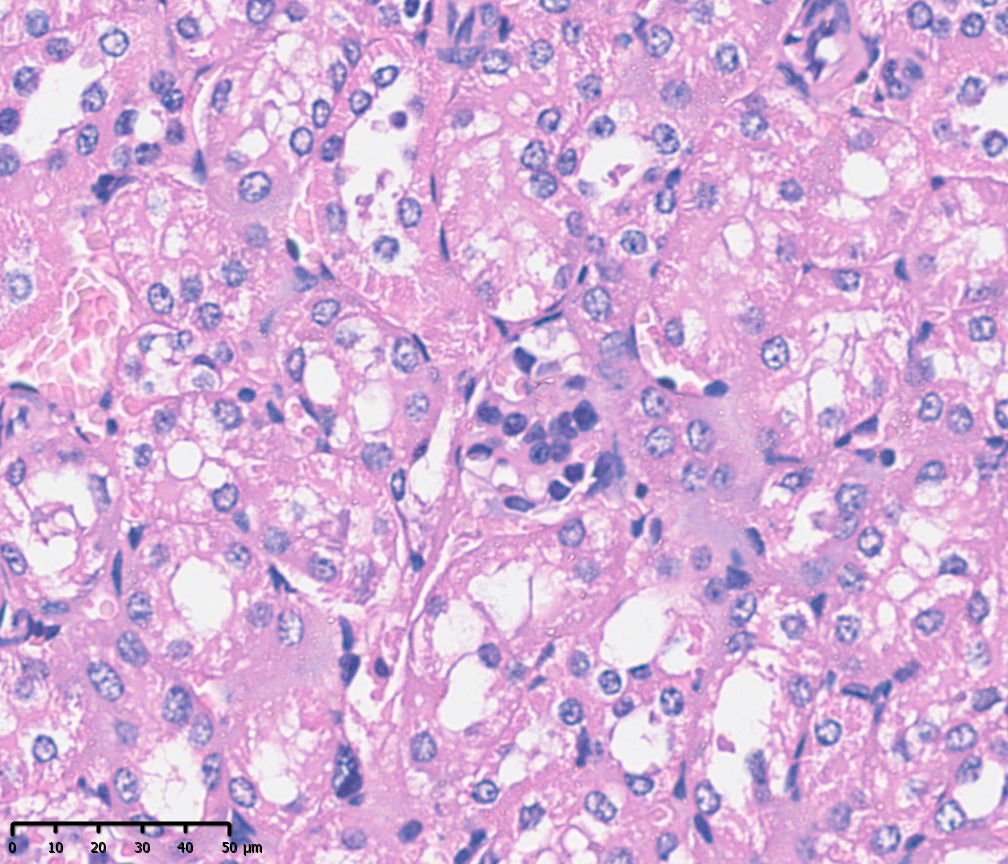

Supplement: Supplementary file 3 — Source data Fig. 2 [file 44319_2024_291_MOESM3_ESM.zip › Figure2G/WT_CLP_KIDNEY.tif]

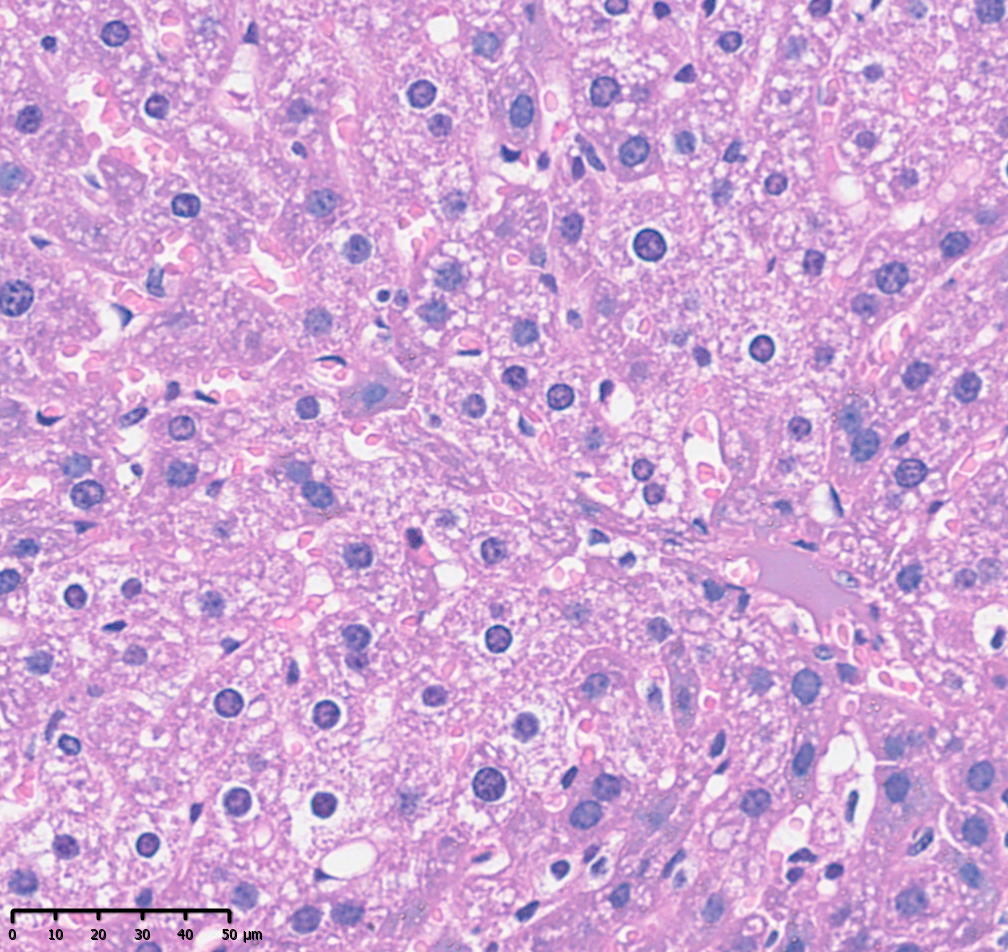

Supplement: Supplementary file 3 — Source data Fig. 2 [file 44319_2024_291_MOESM3_ESM.zip › Figure2G/WT_CLP_LIVER.tif]

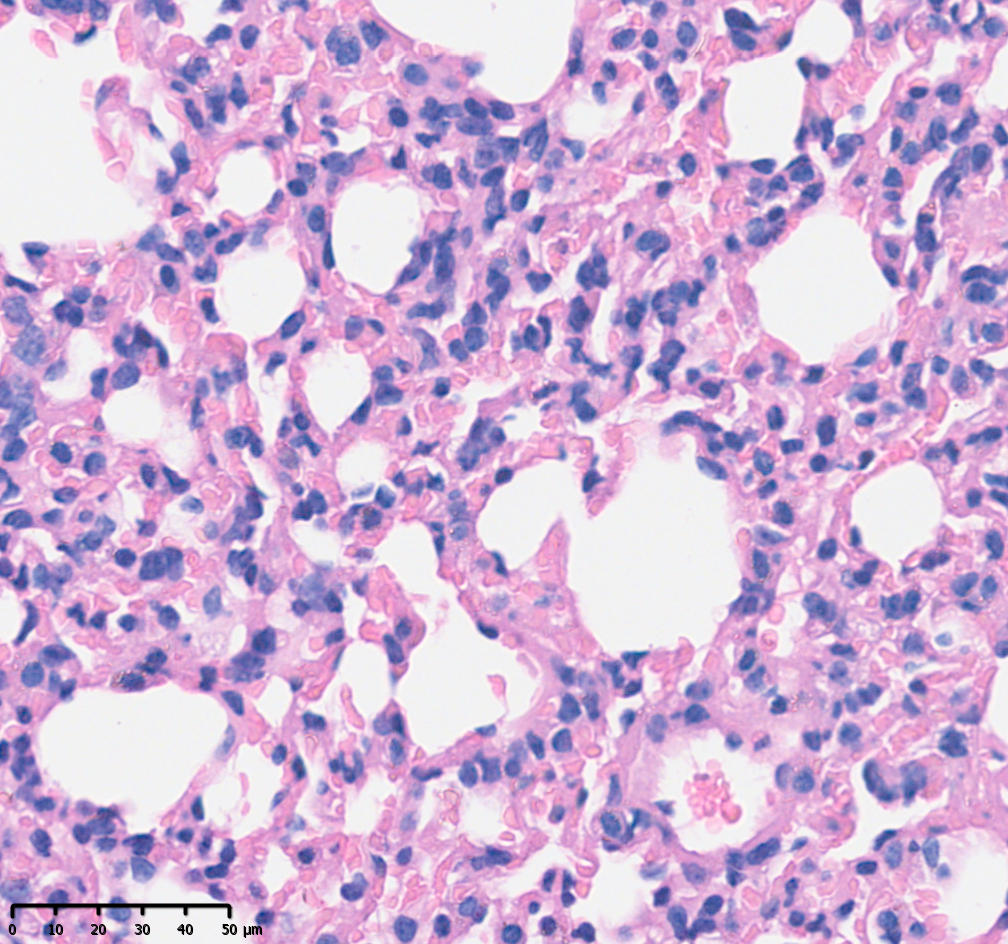

Supplement: Supplementary file 3 — Source data Fig. 2 [file 44319_2024_291_MOESM3_ESM.zip › Figure2G/WT_CLP_LUNG.tif]

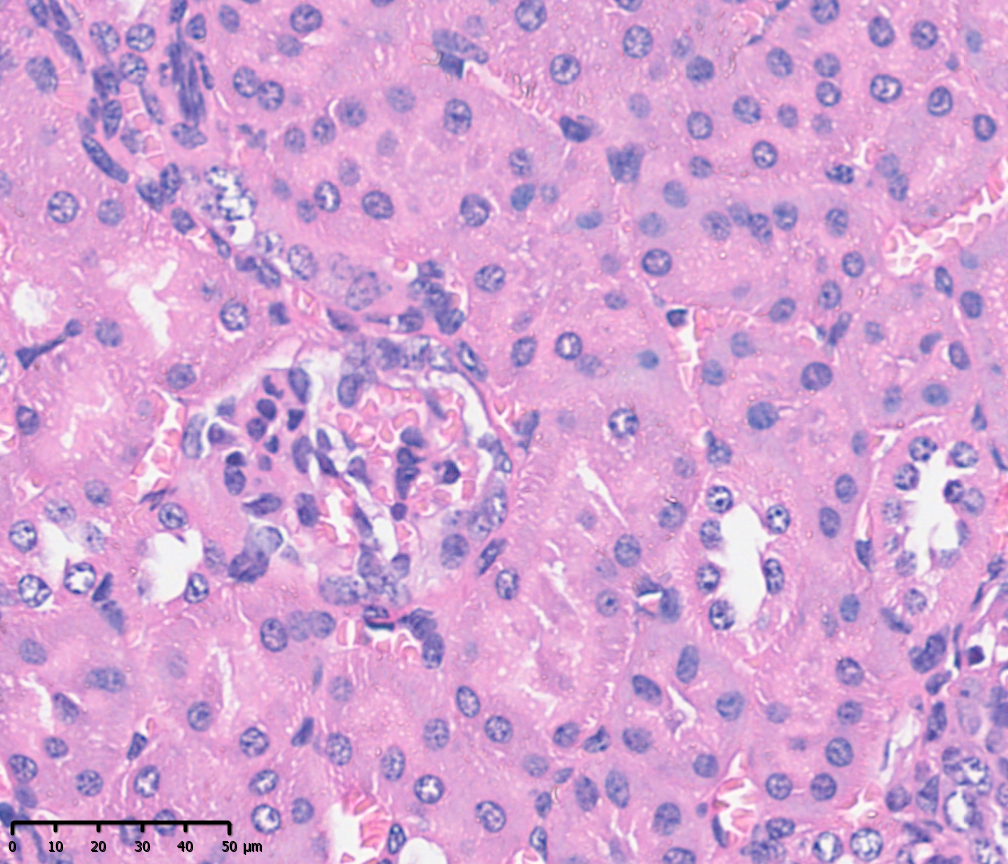

Supplement: Supplementary file 3 — Source data Fig. 2 [file 44319_2024_291_MOESM3_ESM.zip › Figure2G/WT_SHAM_KIDNEY.tif]

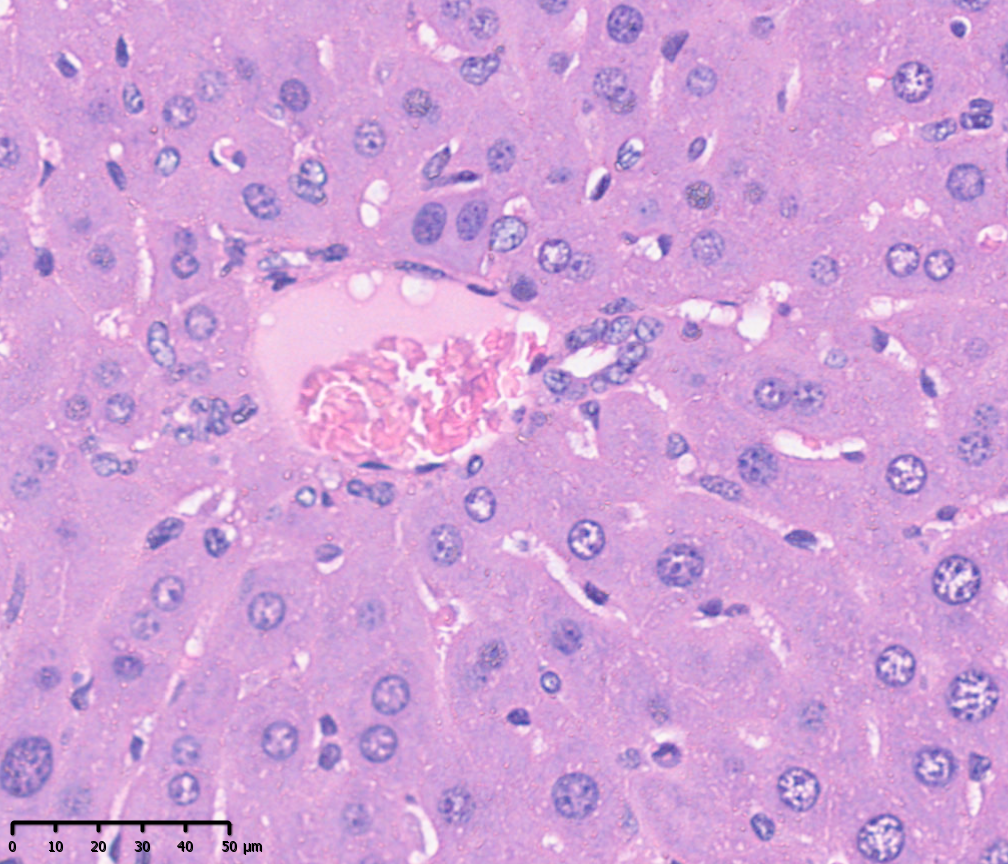

Supplement: Supplementary file 3 — Source data Fig. 2 [file 44319_2024_291_MOESM3_ESM.zip › Figure2G/WT_SHAM_LIVER.tif]

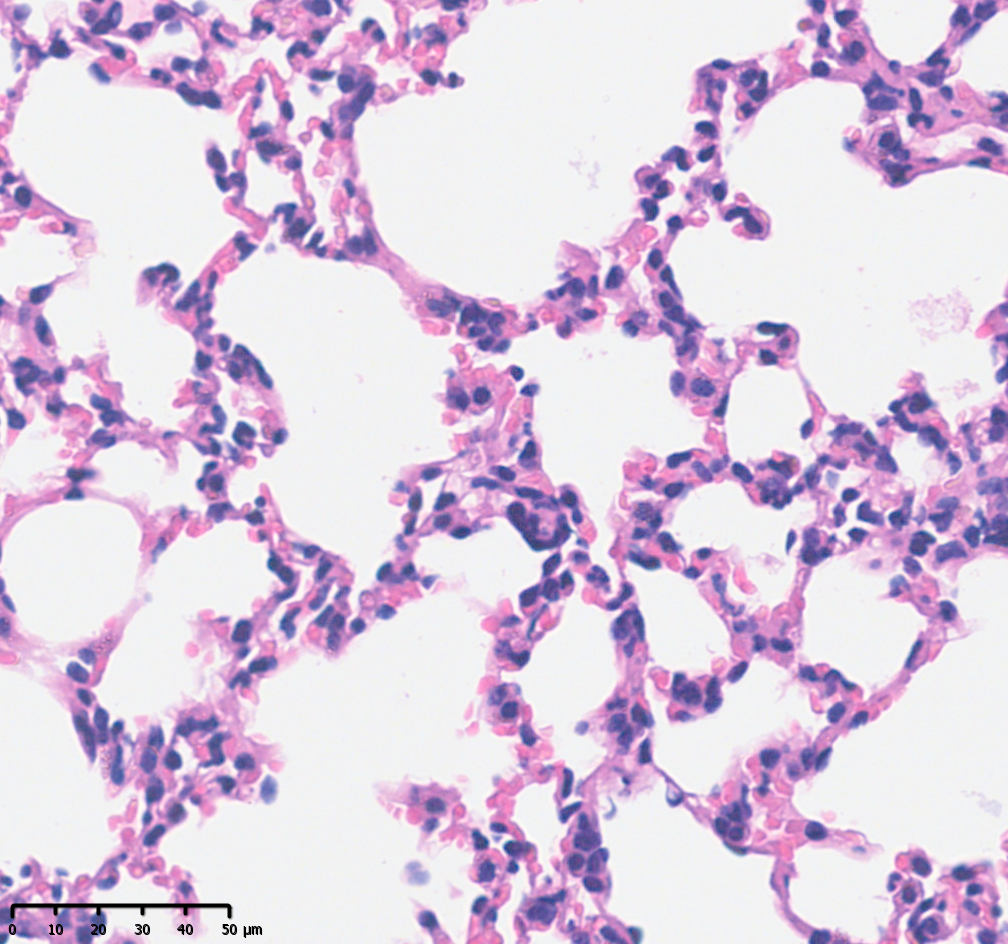

Supplement: Supplementary file 3 — Source data Fig. 2 [file 44319_2024_291_MOESM3_ESM.zip › Figure2G/WT_SHAM_LUNG.tif]

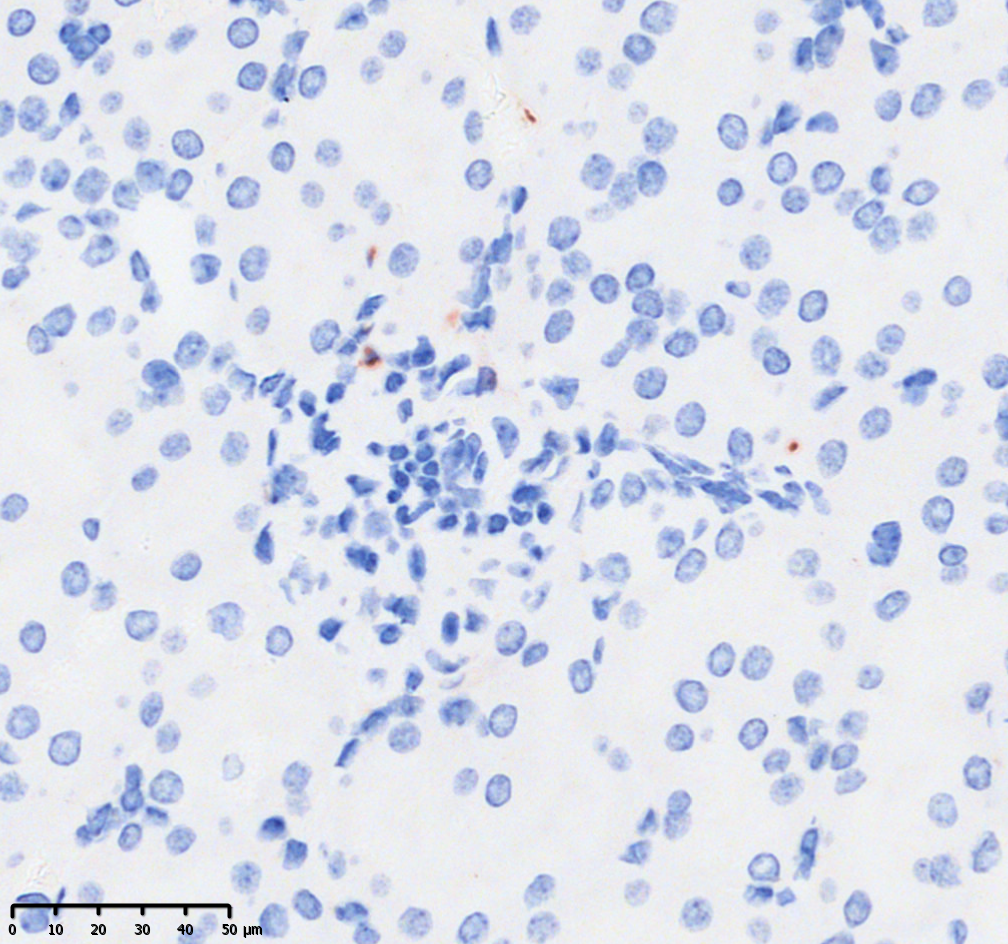

Supplement: Supplementary file 4 — Source data Fig. 3 [file 44319_2024_291_MOESM4_ESM.zip › Figure3B/KO_CLP_KIDNEY.tif]

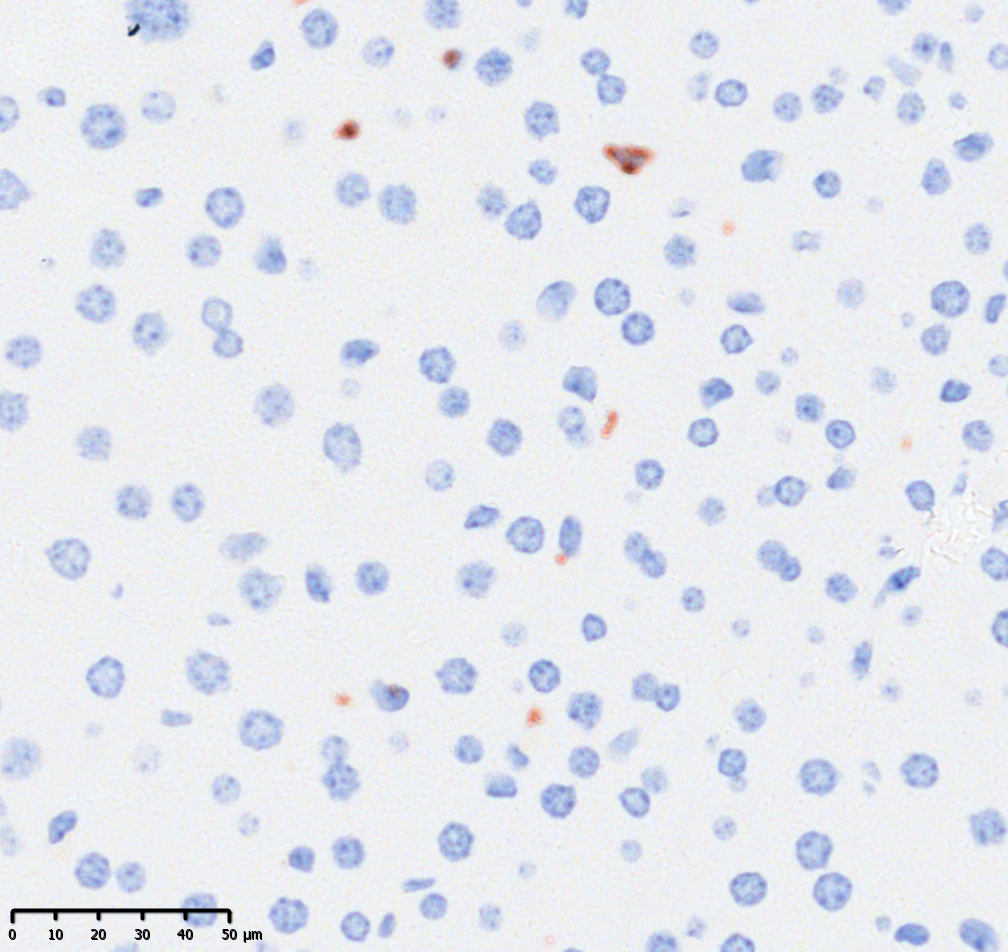

Supplement: Supplementary file 4 — Source data Fig. 3 [file 44319_2024_291_MOESM4_ESM.zip › Figure3B/KO_CLP_LIVER.tif]

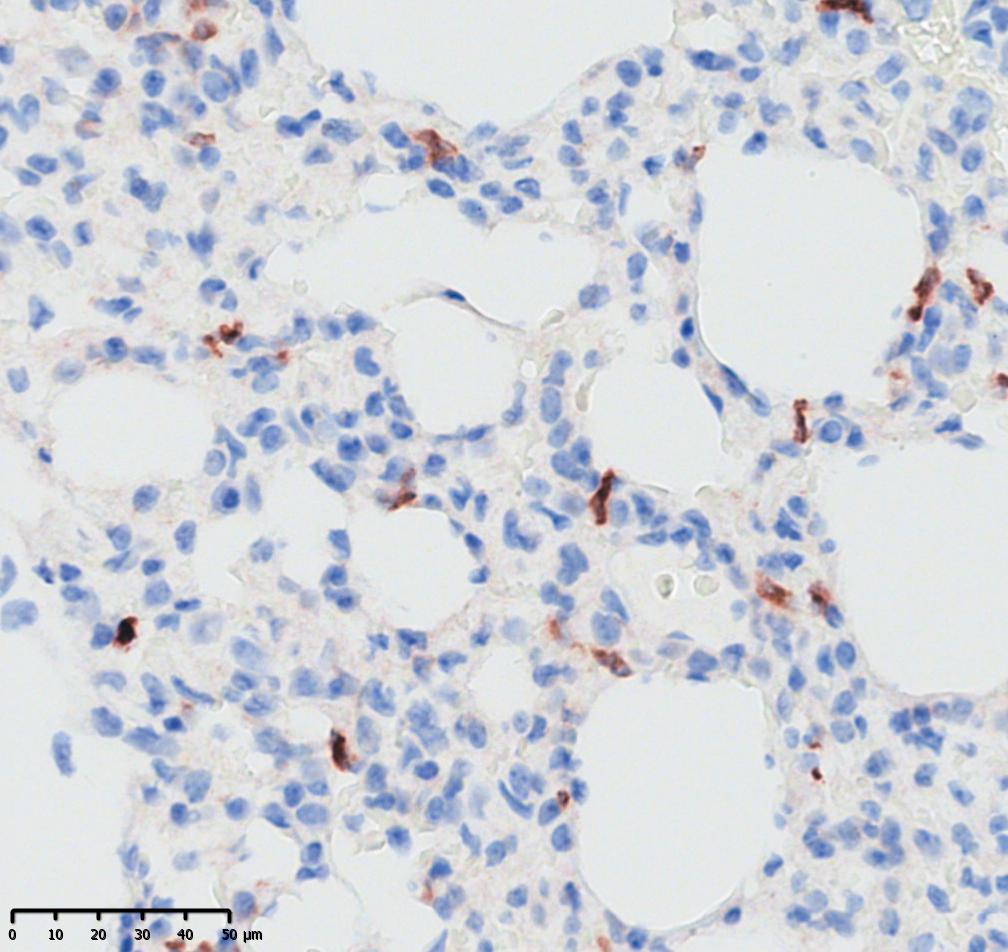

Supplement: Supplementary file 4 — Source data Fig. 3 [file 44319_2024_291_MOESM4_ESM.zip › Figure3B/KO_CLP_LUNG.tif]

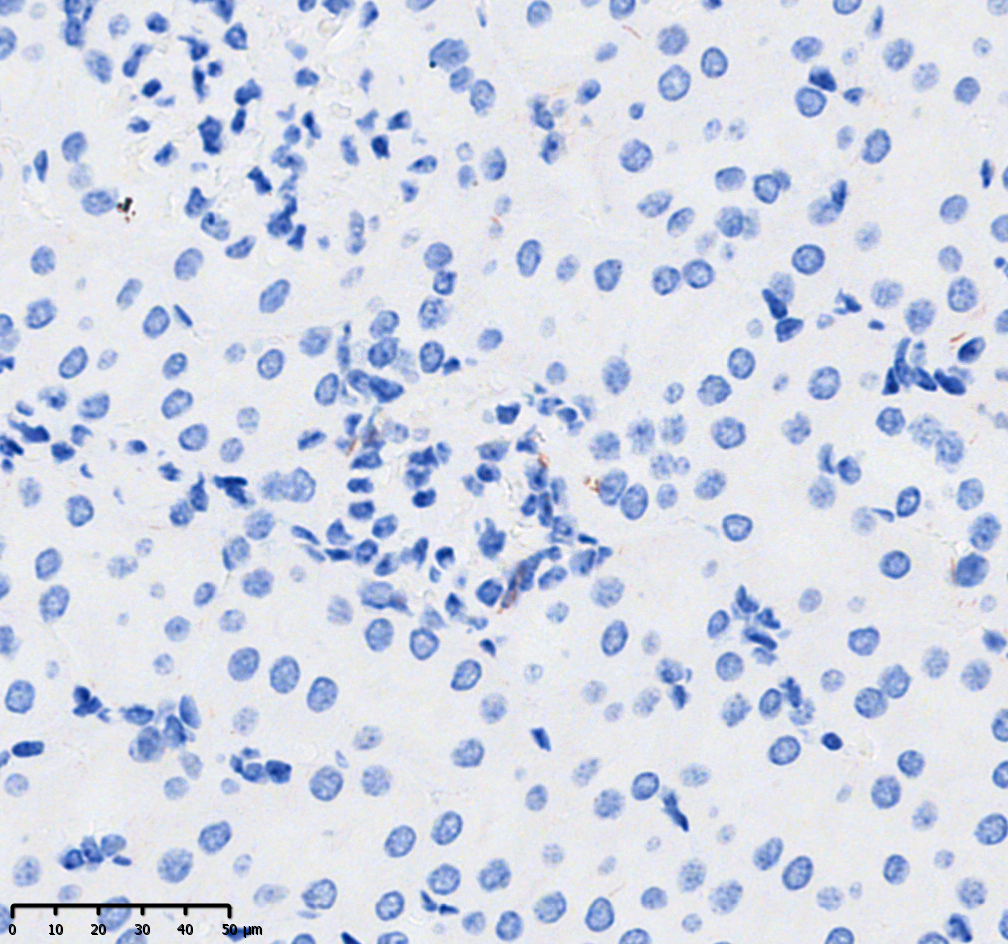

Supplement: Supplementary file 4 — Source data Fig. 3 [file 44319_2024_291_MOESM4_ESM.zip › Figure3B/KO_SHAM_KIDNEY.tif]

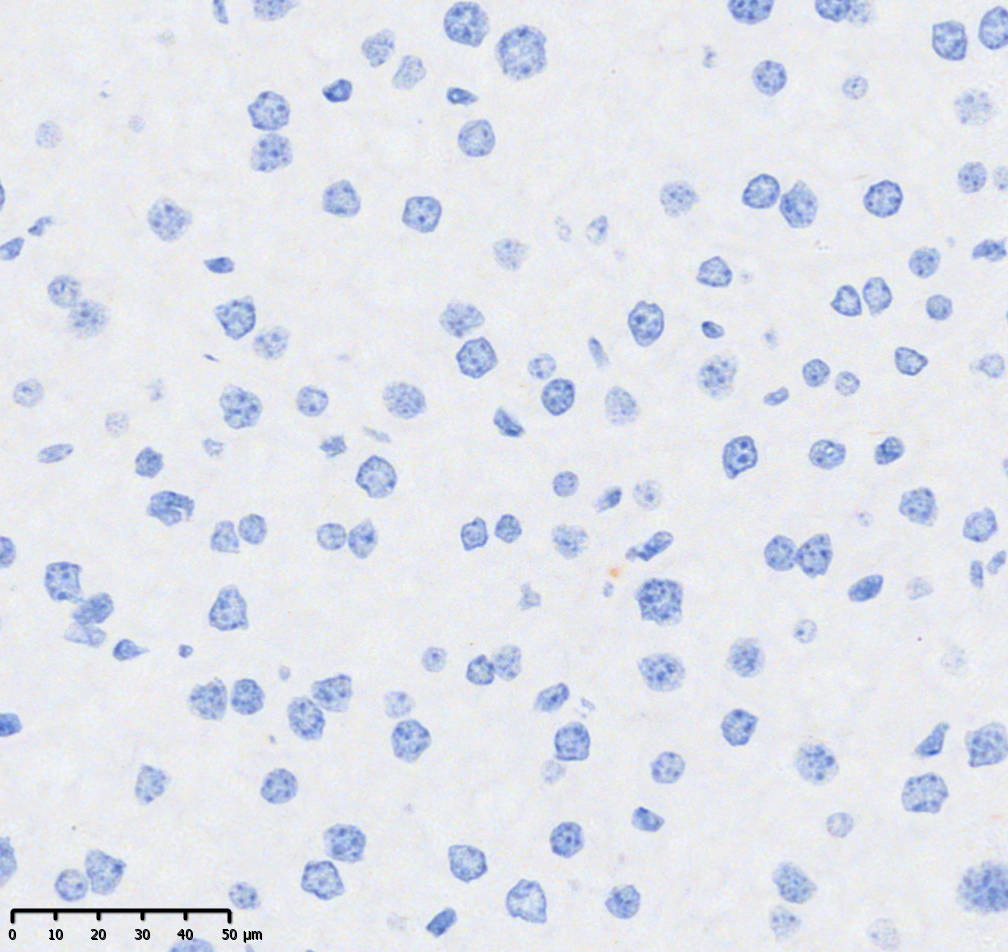

Supplement: Supplementary file 4 — Source data Fig. 3 [file 44319_2024_291_MOESM4_ESM.zip › Figure3B/KO_SHAM_LIVER.tif]

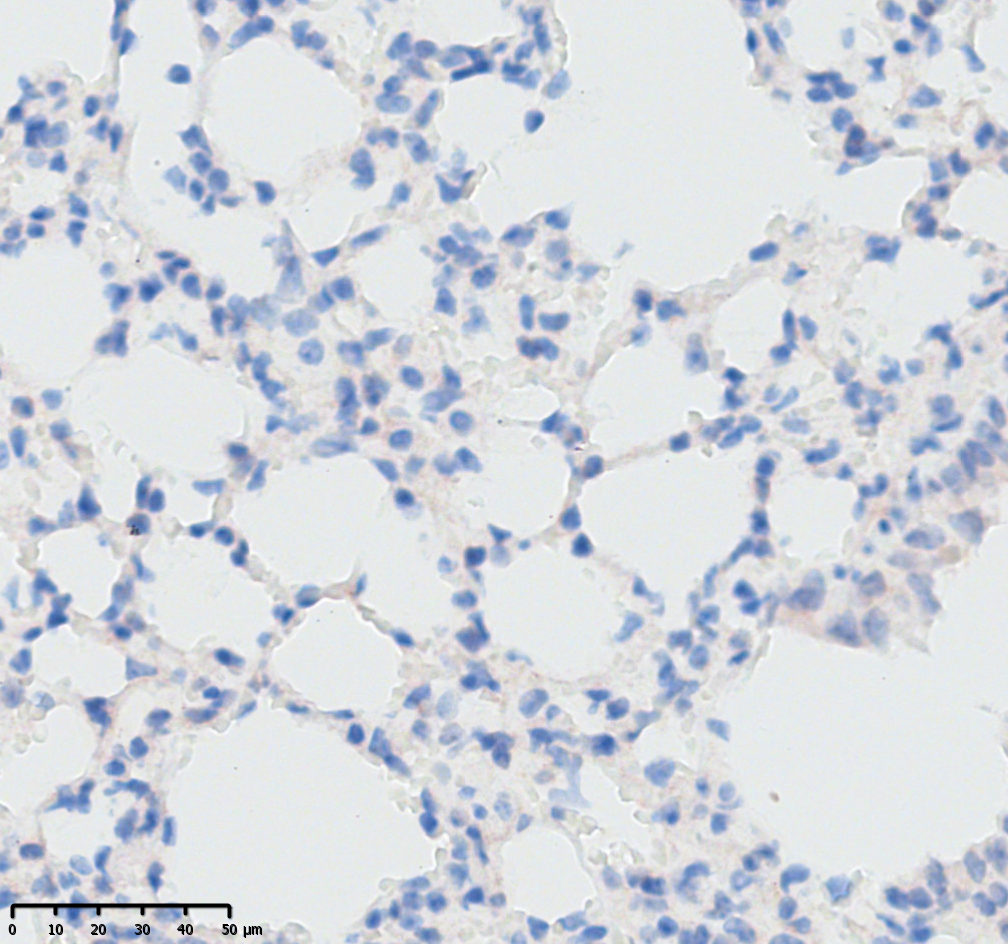

Supplement: Supplementary file 4 — Source data Fig. 3 [file 44319_2024_291_MOESM4_ESM.zip › Figure3B/KO_SHAM_LUNG.tif]

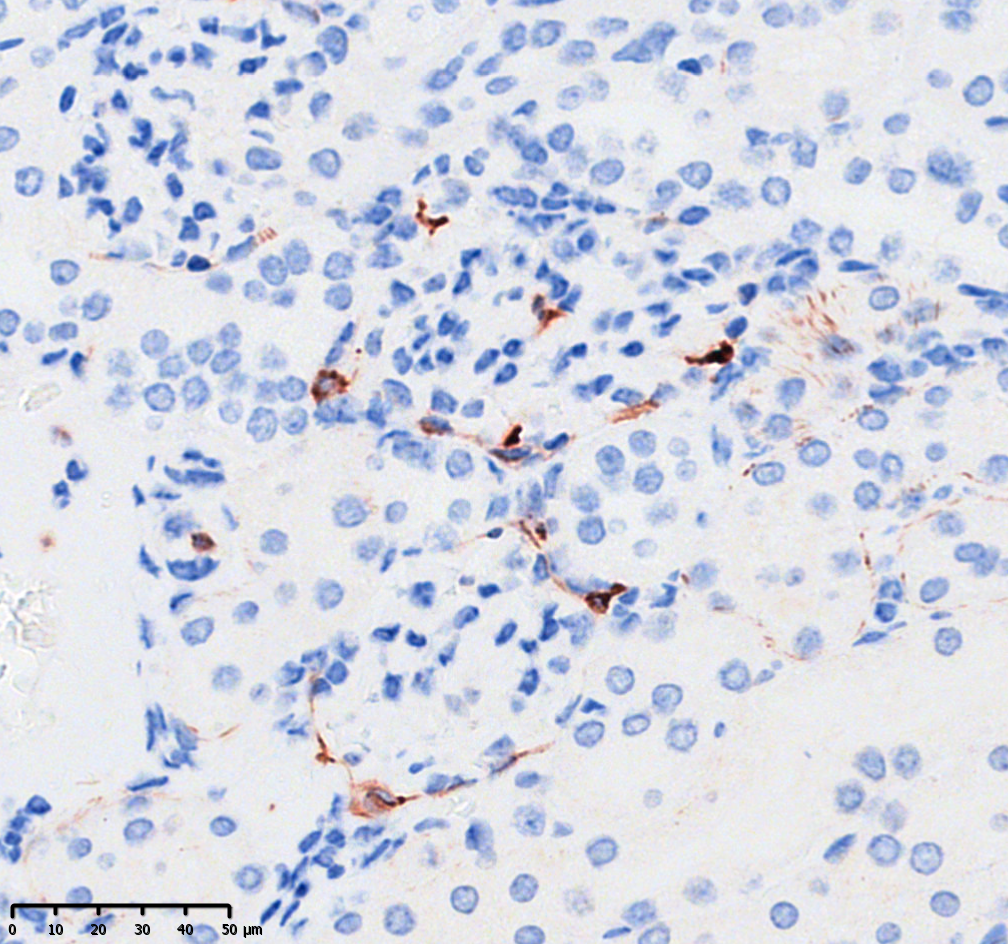

Supplement: Supplementary file 4 — Source data Fig. 3 [file 44319_2024_291_MOESM4_ESM.zip › Figure3B/WT_CLP_KIDNEY.tif]

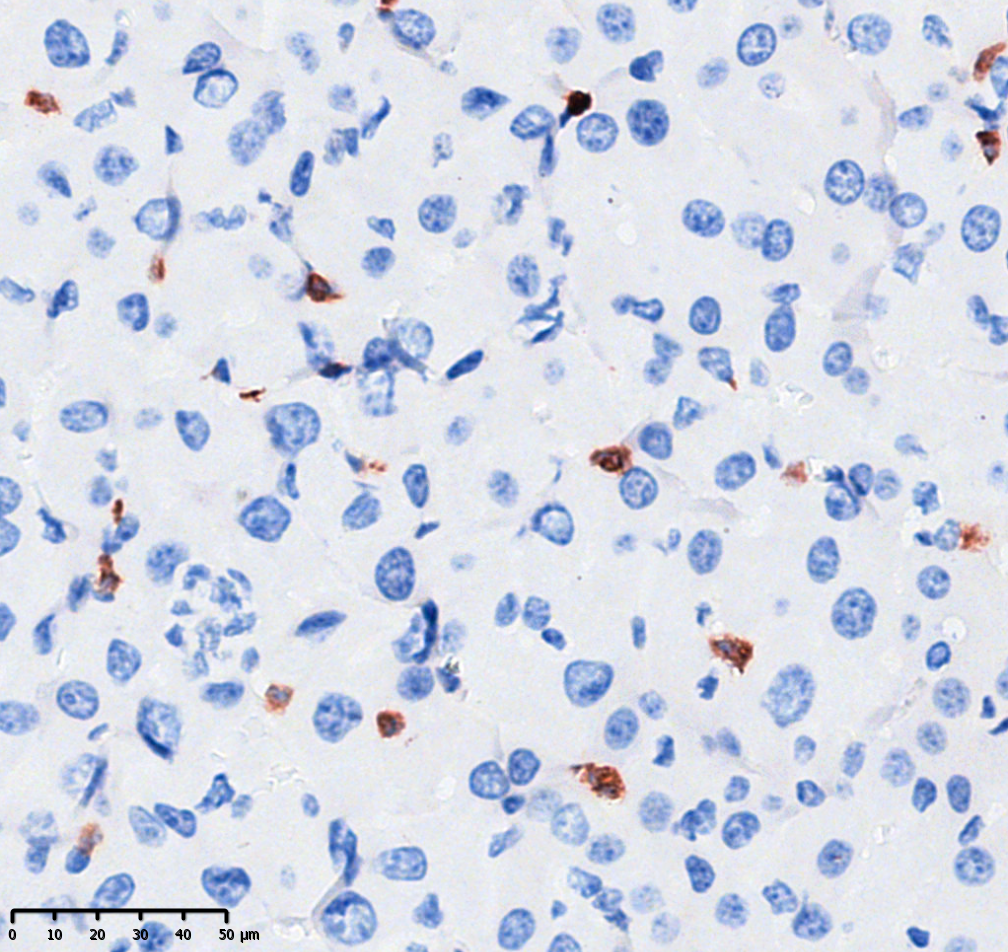

Supplement: Supplementary file 4 — Source data Fig. 3 [file 44319_2024_291_MOESM4_ESM.zip › Figure3B/WT_CLP_LIVER.tif]

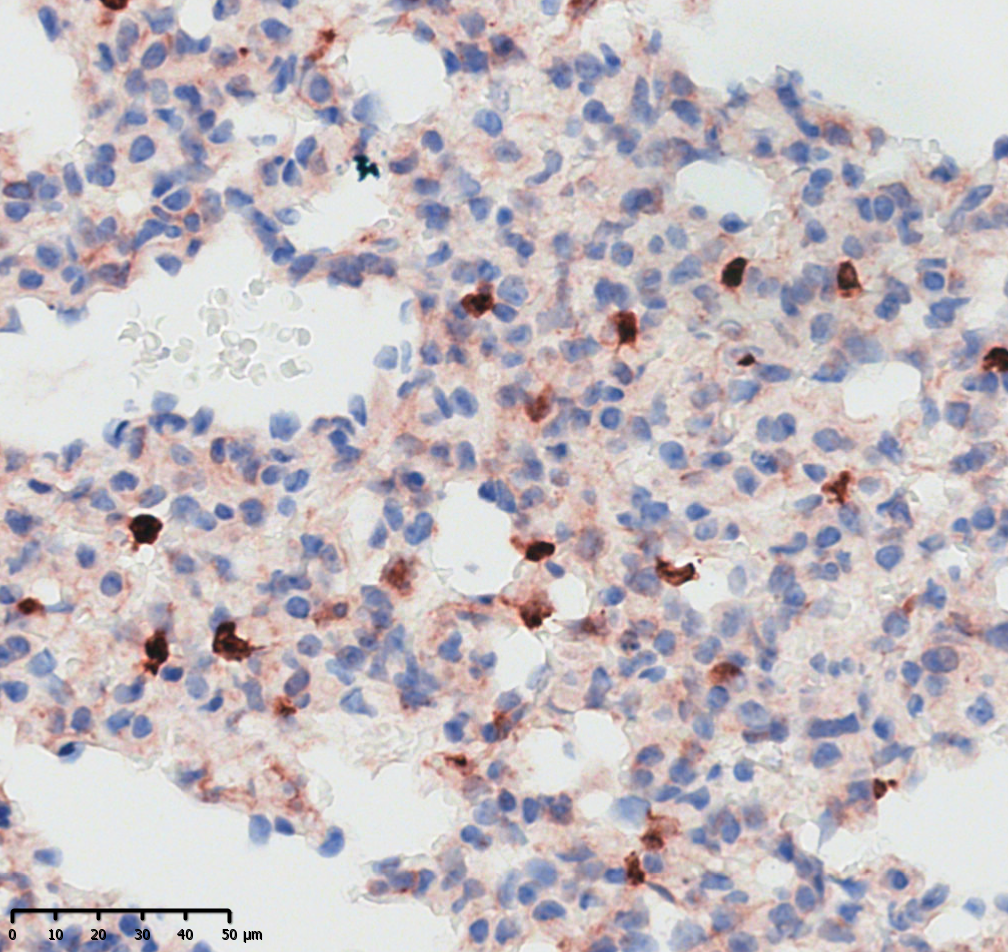

Supplement: Supplementary file 4 — Source data Fig. 3 [file 44319_2024_291_MOESM4_ESM.zip › Figure3B/WT_CLP_LUNG.tif]

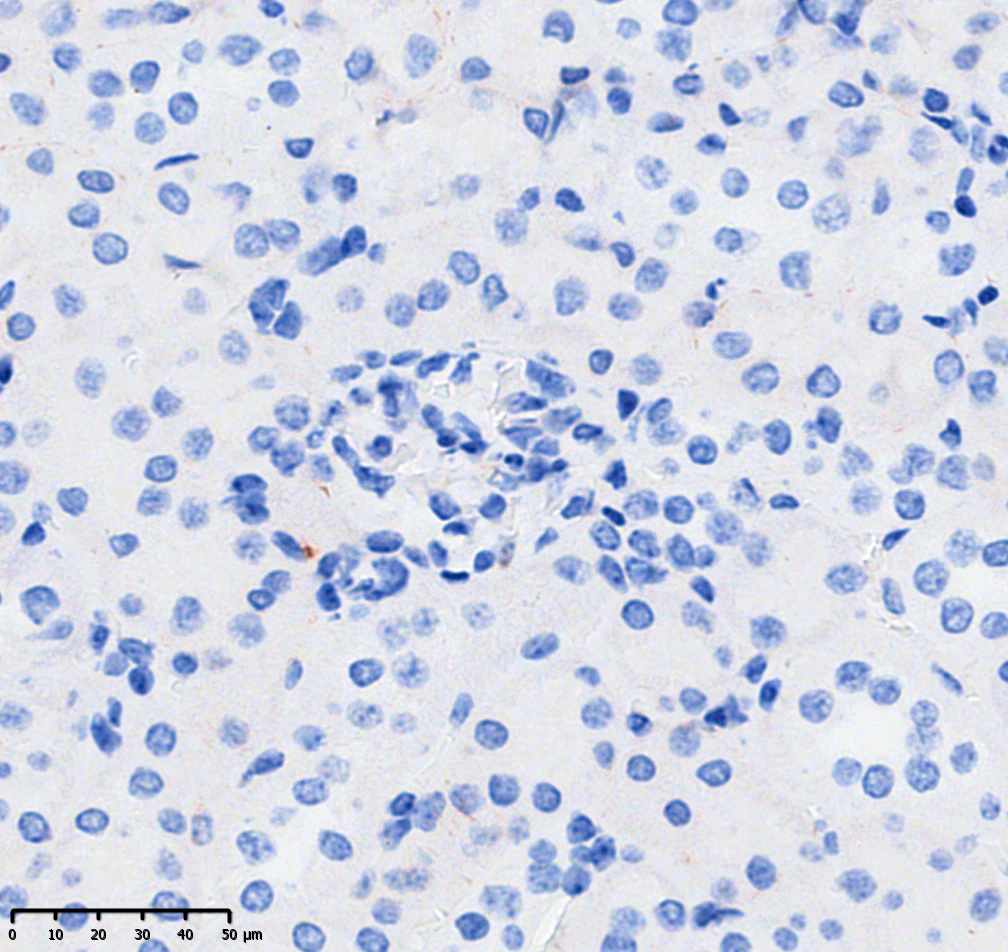

Supplement: Supplementary file 4 — Source data Fig. 3 [file 44319_2024_291_MOESM4_ESM.zip › Figure3B/WT_SHAM_KIDNEY.tif]

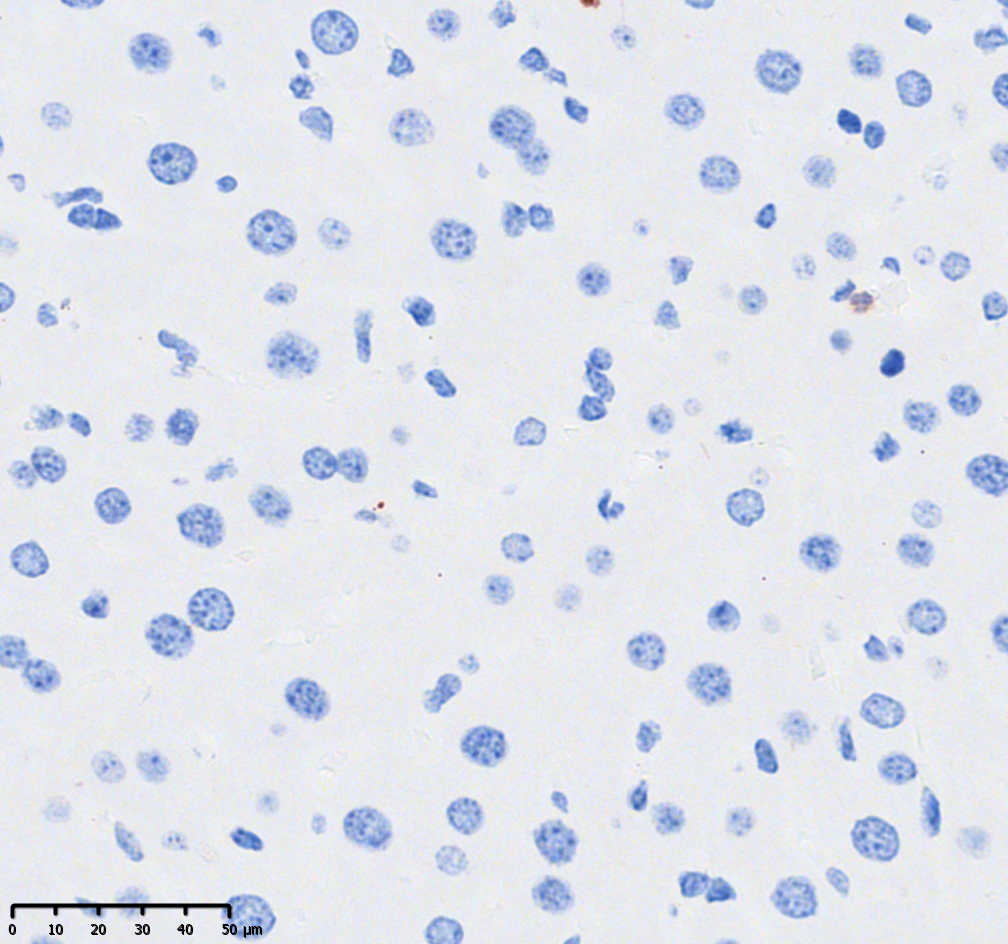

Supplement: Supplementary file 4 — Source data Fig. 3 [file 44319_2024_291_MOESM4_ESM.zip › Figure3B/WT_SHAM_LIVER.tif]

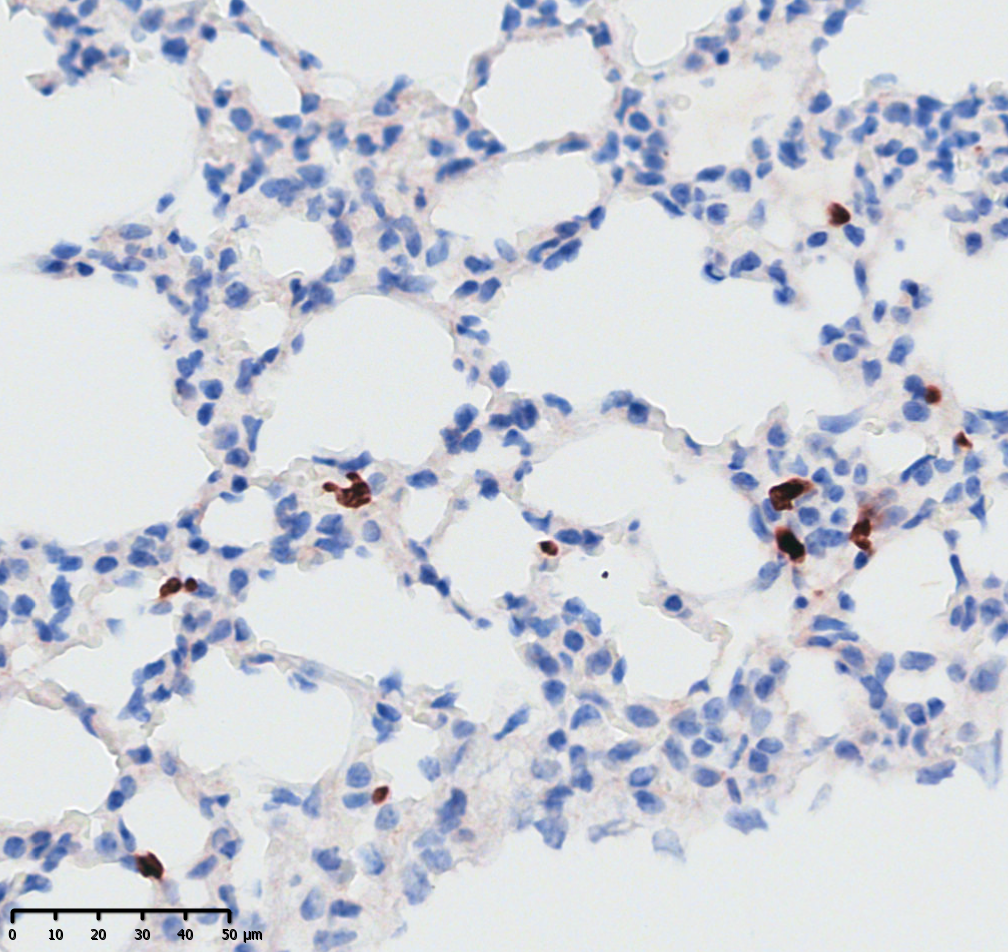

Supplement: Supplementary file 4 — Source data Fig. 3 [file 44319_2024_291_MOESM4_ESM.zip › Figure3B/WT_SHAM_LUNG.tif]

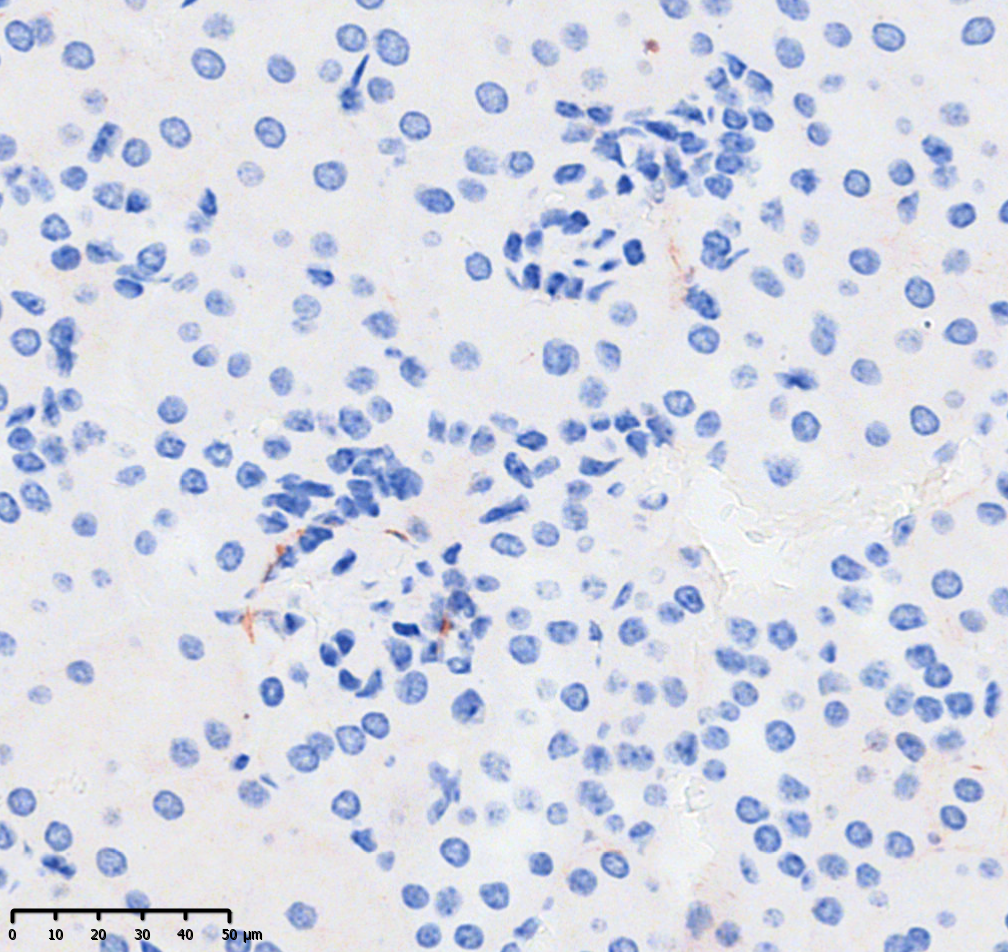

Supplement: Supplementary file 5 — Source data Fig. 4 [file 44319_2024_291_MOESM5_ESM.zip › Figure4B/KO_KIDNEY.tif]

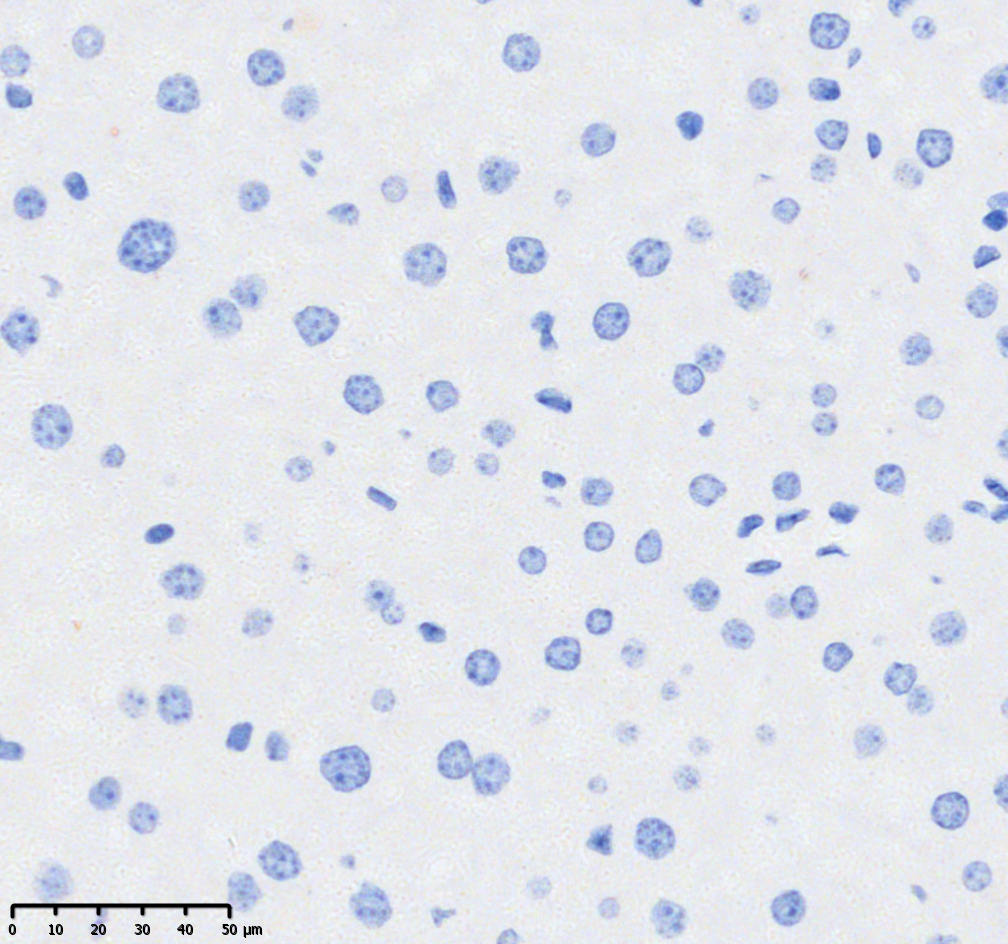

Supplement: Supplementary file 5 — Source data Fig. 4 [file 44319_2024_291_MOESM5_ESM.zip › Figure4B/KO_LIVER.tif]

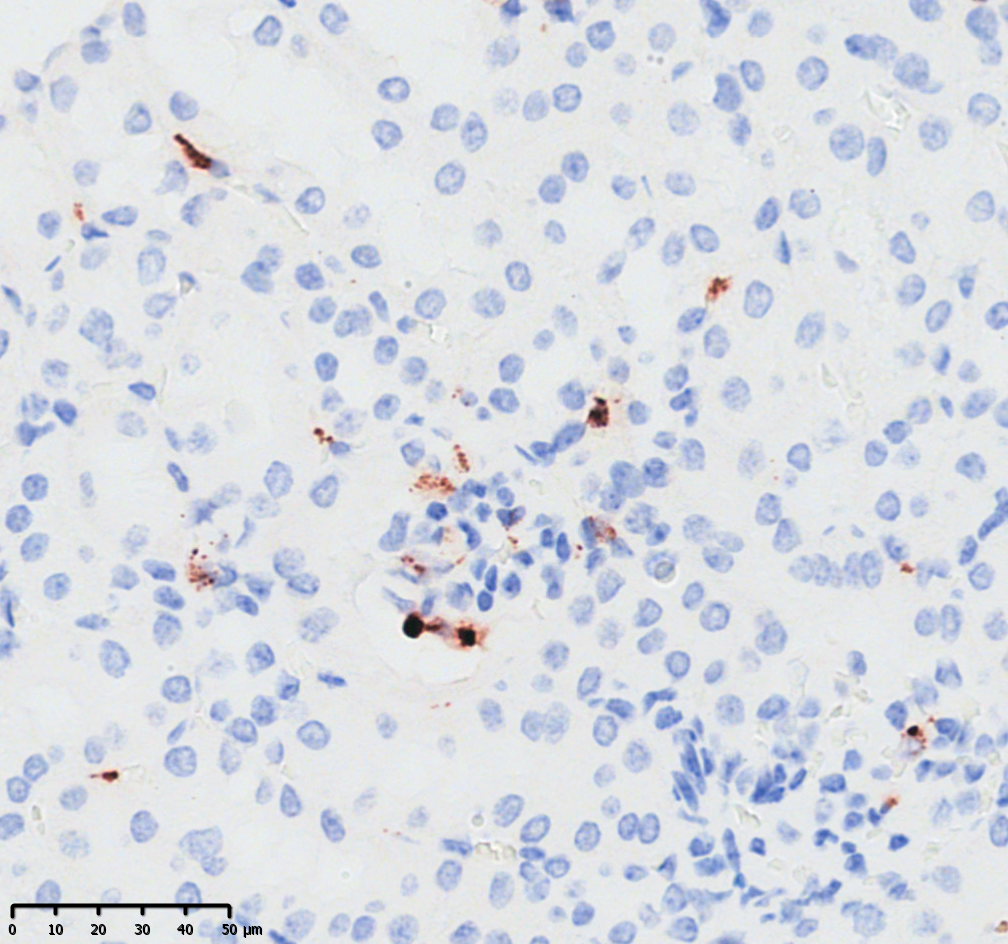

Supplement: Supplementary file 5 — Source data Fig. 4 [file 44319_2024_291_MOESM5_ESM.zip › Figure4B/KO_LPS_KIDNEY.tif]

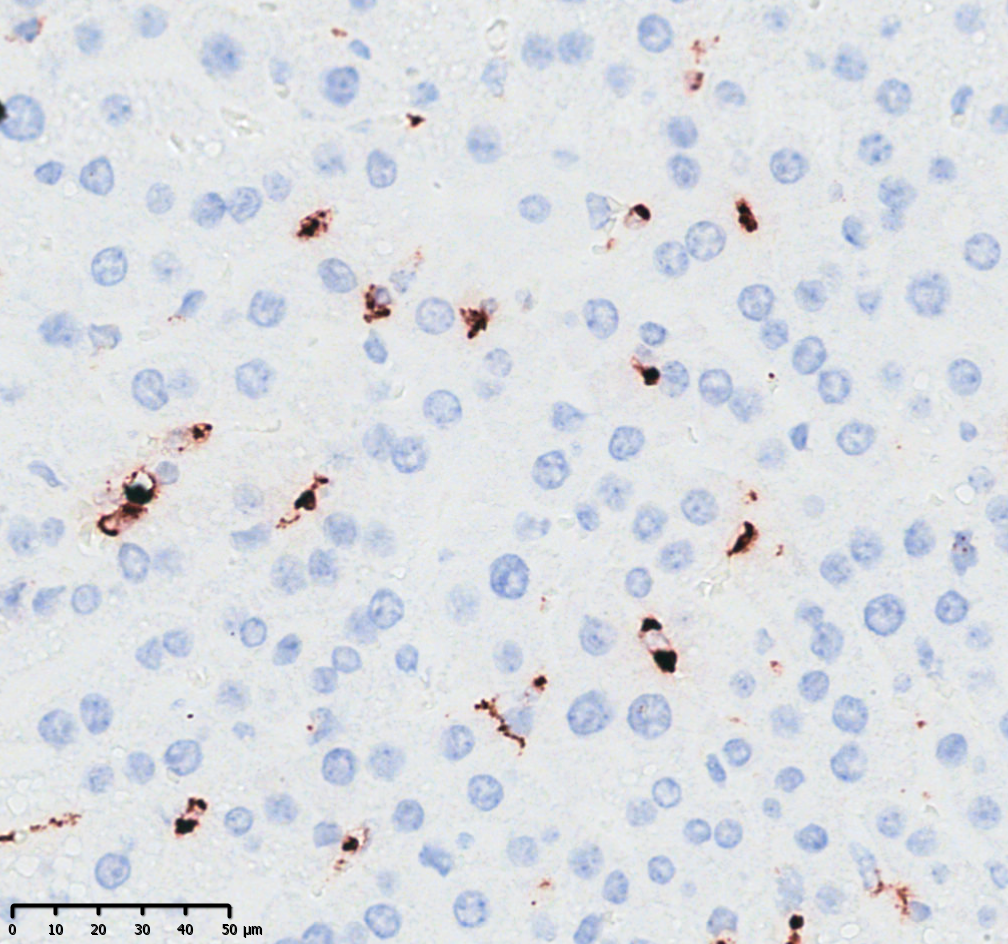

Supplement: Supplementary file 5 — Source data Fig. 4 [file 44319_2024_291_MOESM5_ESM.zip › Figure4B/KO_LPS_LIVER.tif]

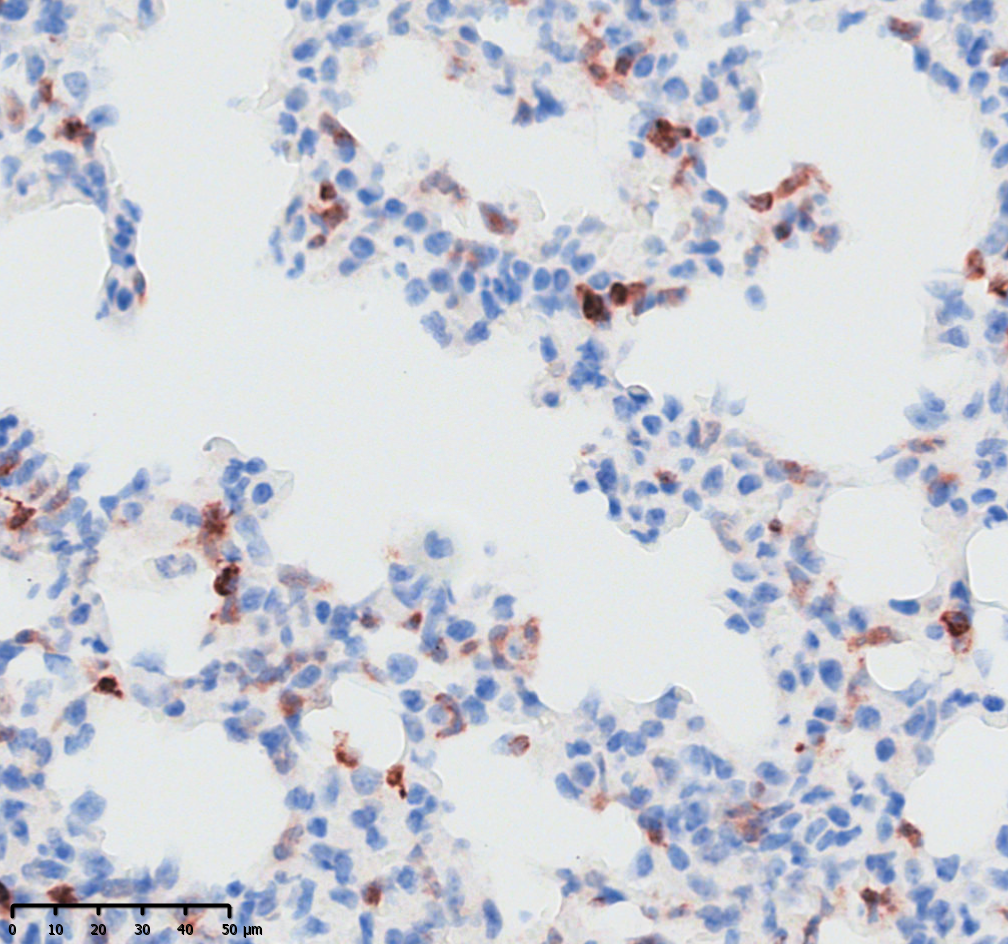

Supplement: Supplementary file 5 — Source data Fig. 4 [file 44319_2024_291_MOESM5_ESM.zip › Figure4B/KO_LPS_LUNG.tif]

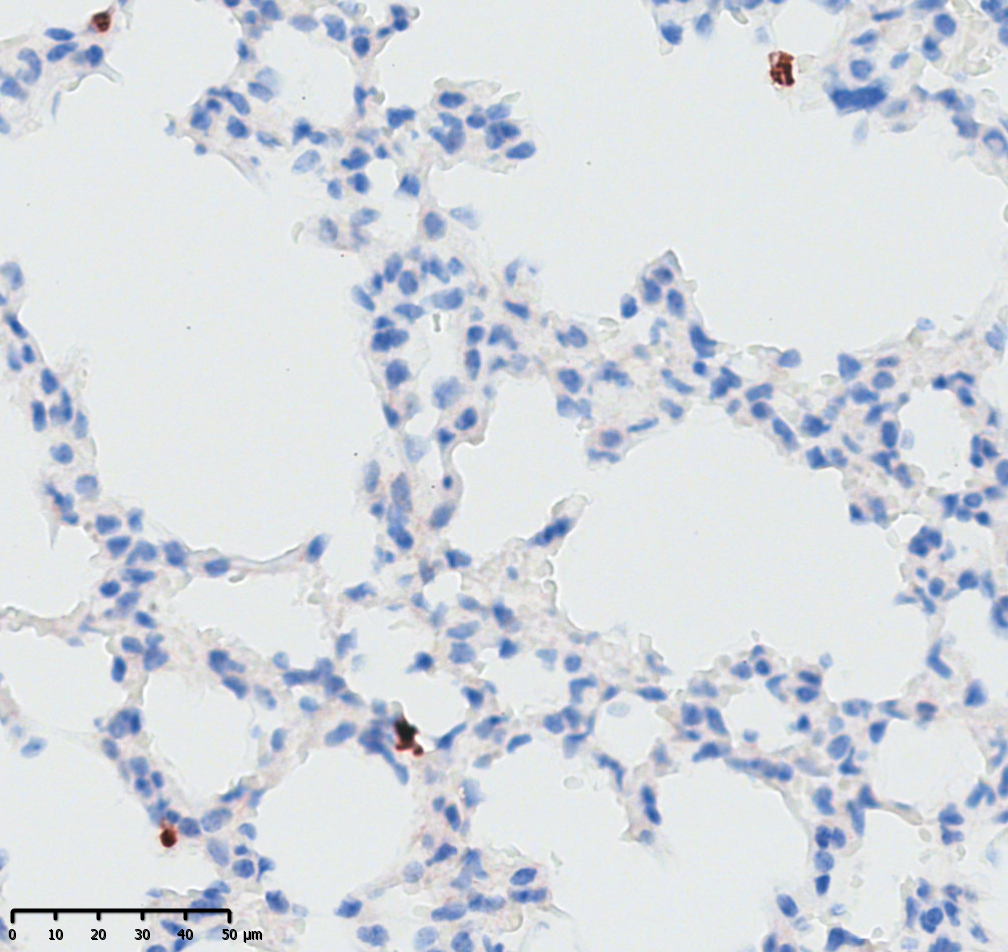

Supplement: Supplementary file 5 — Source data Fig. 4 [file 44319_2024_291_MOESM5_ESM.zip › Figure4B/KO_LUNG.tif]

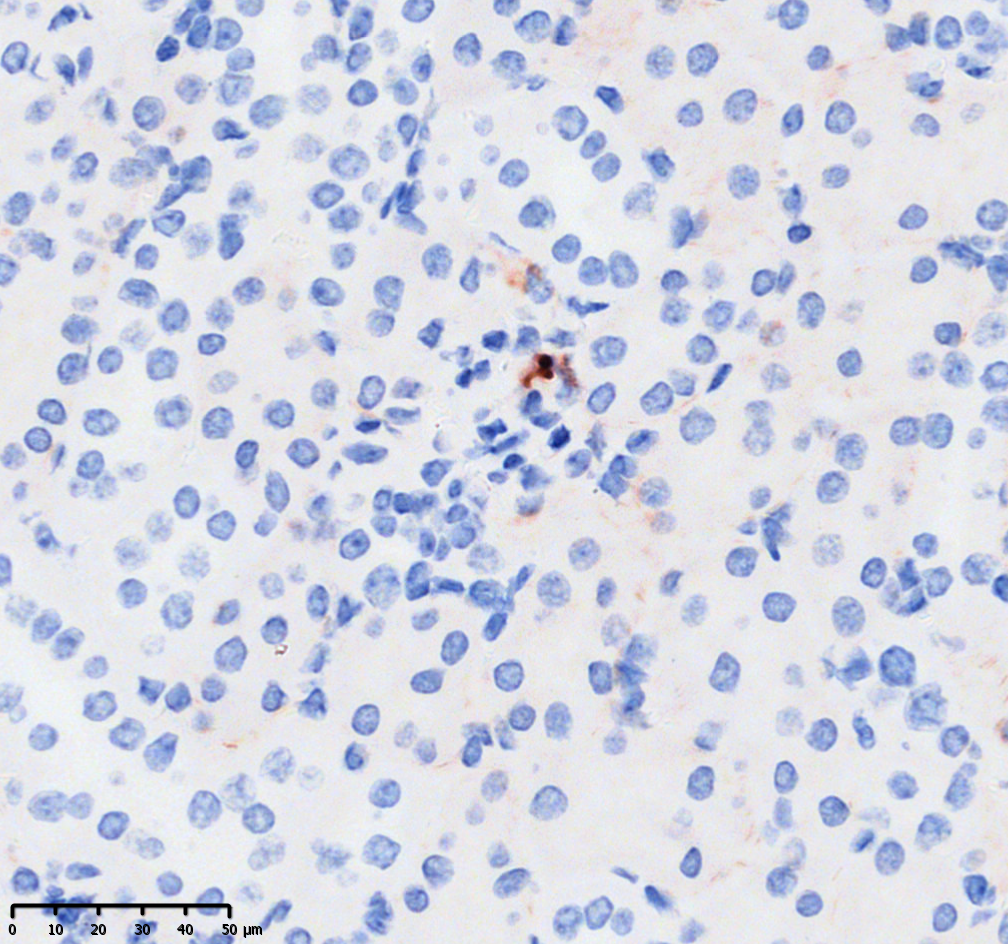

Supplement: Supplementary file 5 — Source data Fig. 4 [file 44319_2024_291_MOESM5_ESM.zip › Figure4B/WT_KIDNEY.tif]

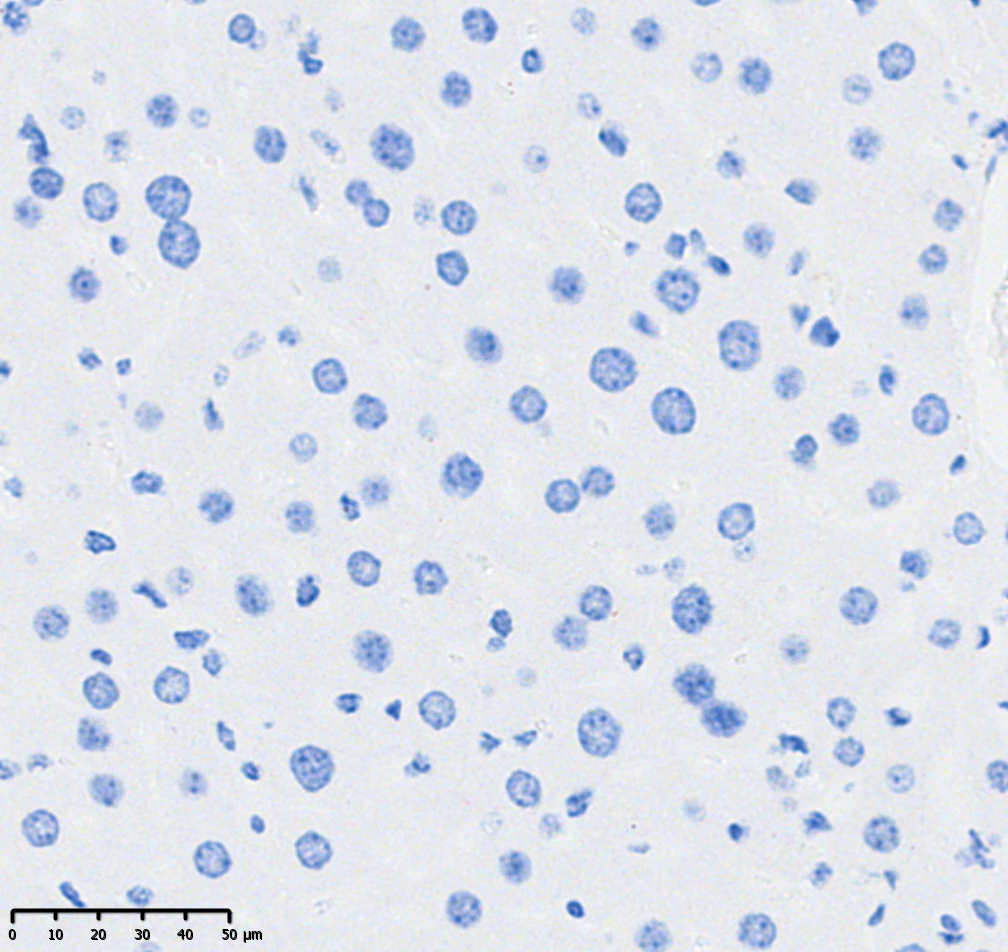

Supplement: Supplementary file 5 — Source data Fig. 4 [file 44319_2024_291_MOESM5_ESM.zip › Figure4B/WT_LIVER.tif]

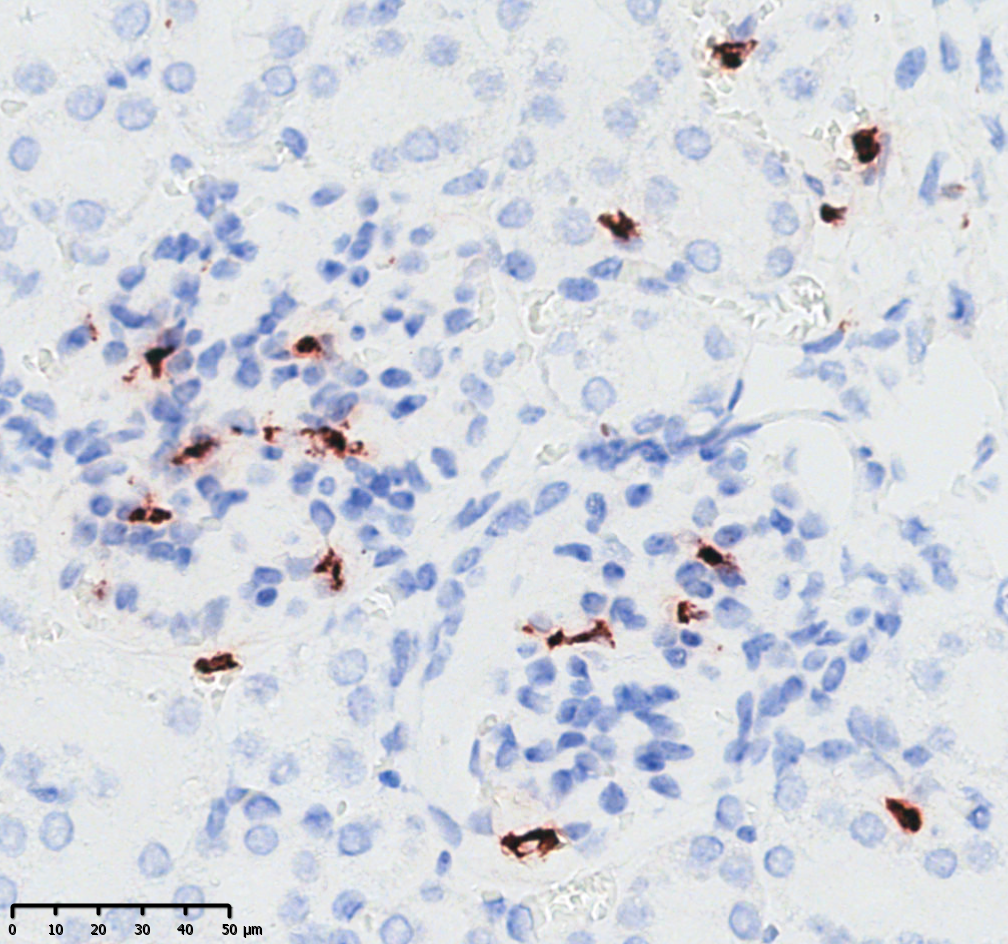

Supplement: Supplementary file 5 — Source data Fig. 4 [file 44319_2024_291_MOESM5_ESM.zip › Figure4B/WT_LPS_KIDNEY.tif]

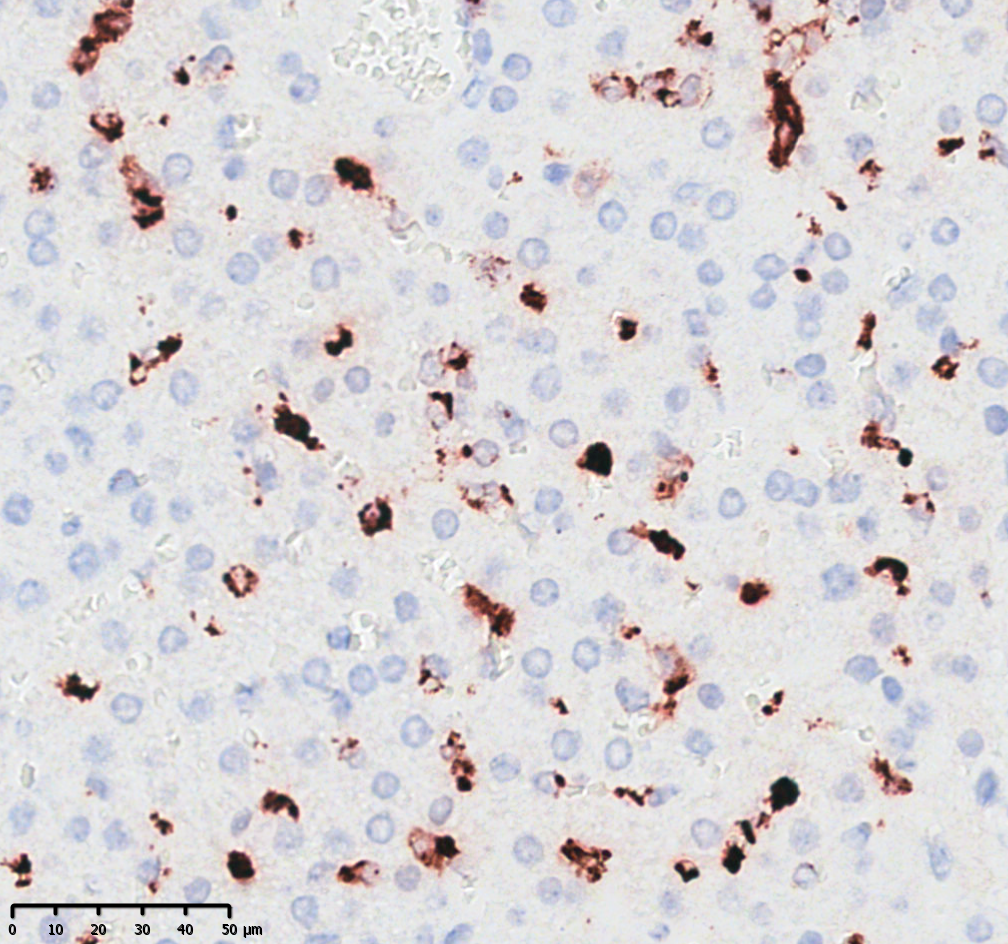

Supplement: Supplementary file 5 — Source data Fig. 4 [file 44319_2024_291_MOESM5_ESM.zip › Figure4B/WT_LPS_LIVER.tif]

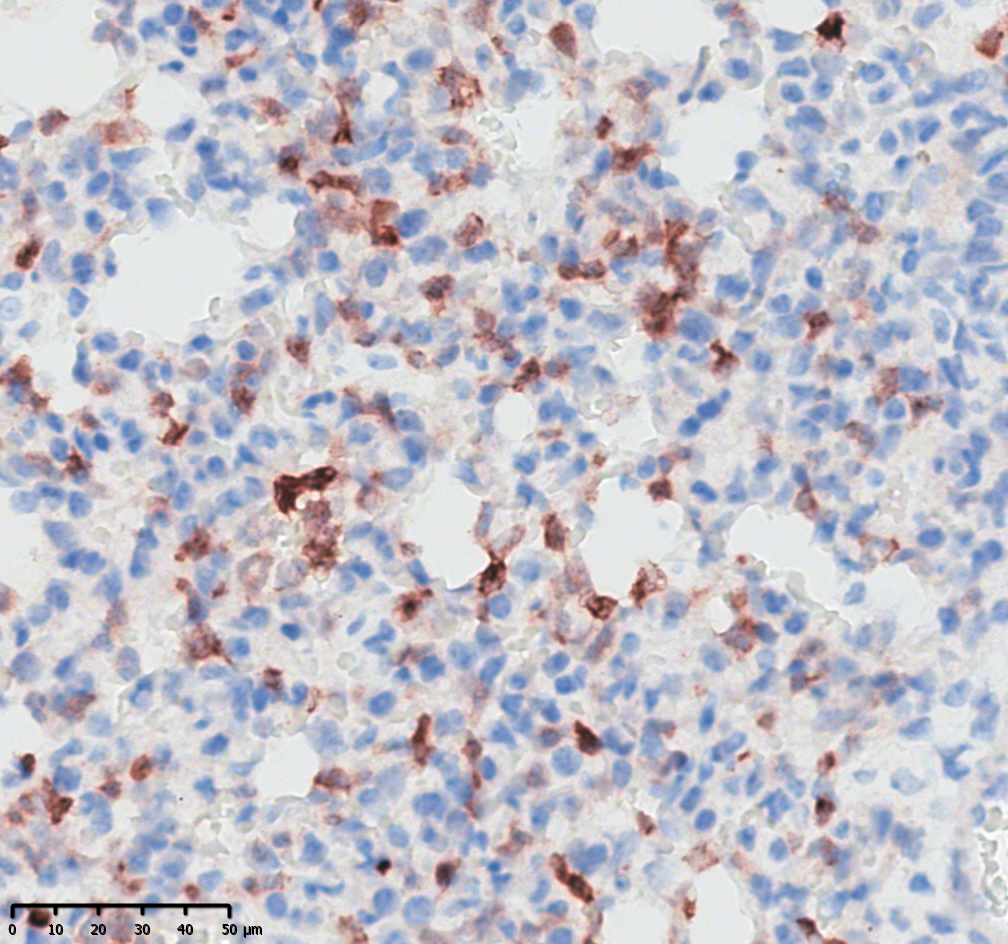

Supplement: Supplementary file 5 — Source data Fig. 4 [file 44319_2024_291_MOESM5_ESM.zip › Figure4B/WT_LPS_LUNG.tif]

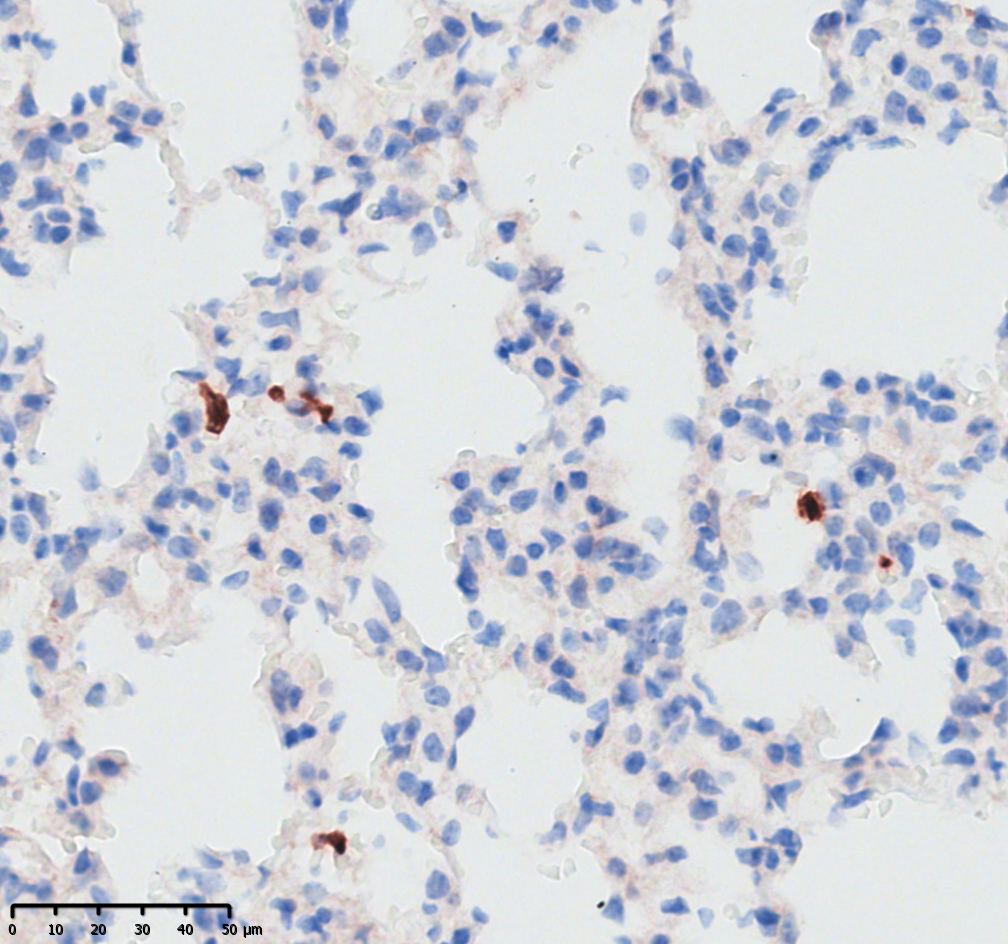

Supplement: Supplementary file 5 — Source data Fig. 4 [file 44319_2024_291_MOESM5_ESM.zip › Figure4B/WT_LUNG.tif]

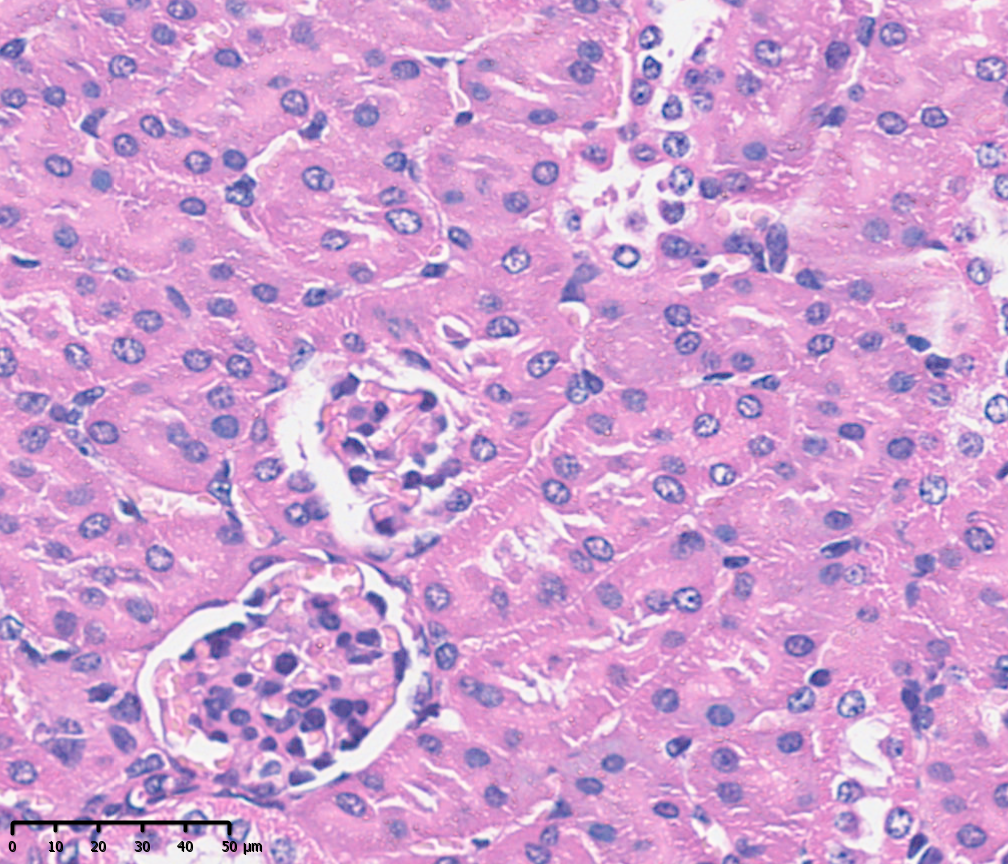

Supplement: Supplementary file 8 — Source data Fig. 7 [file 44319_2024_291_MOESM8_ESM.zip › Figure7G/KO_KIDNEY.tif]

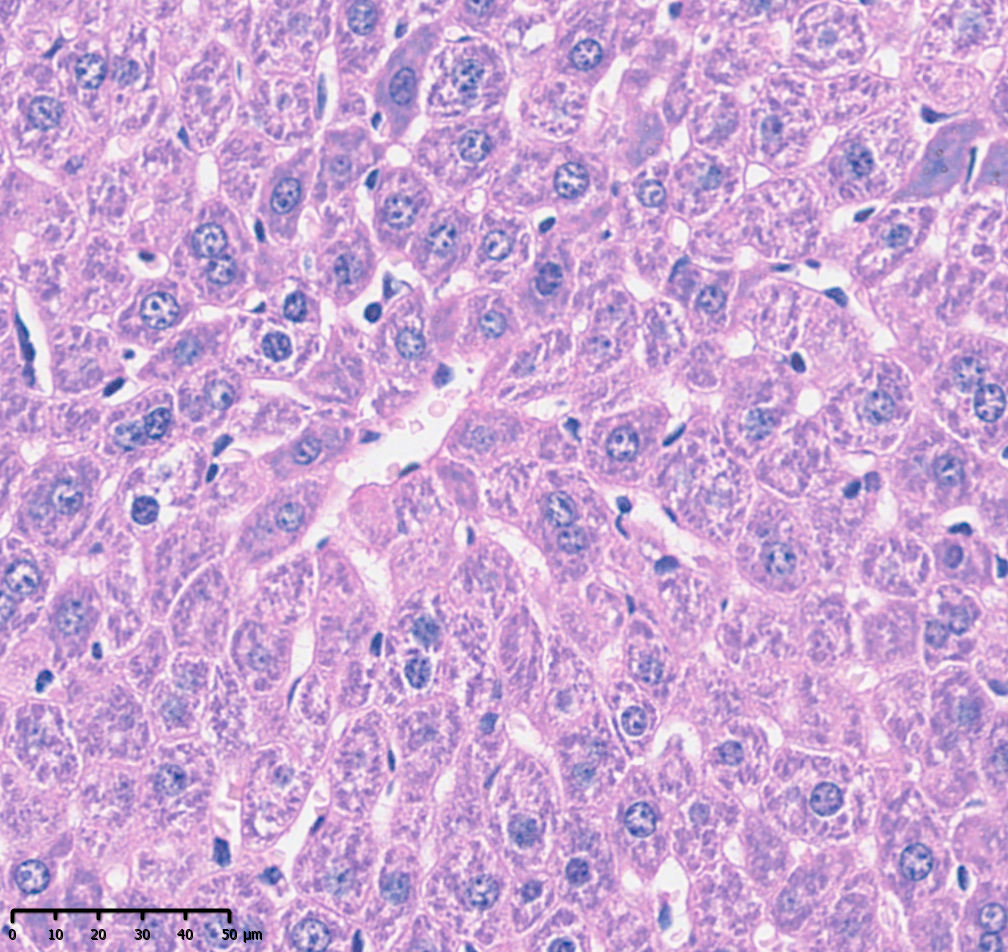

Supplement: Supplementary file 8 — Source data Fig. 7 [file 44319_2024_291_MOESM8_ESM.zip › Figure7G/KO_LIVER.tif]

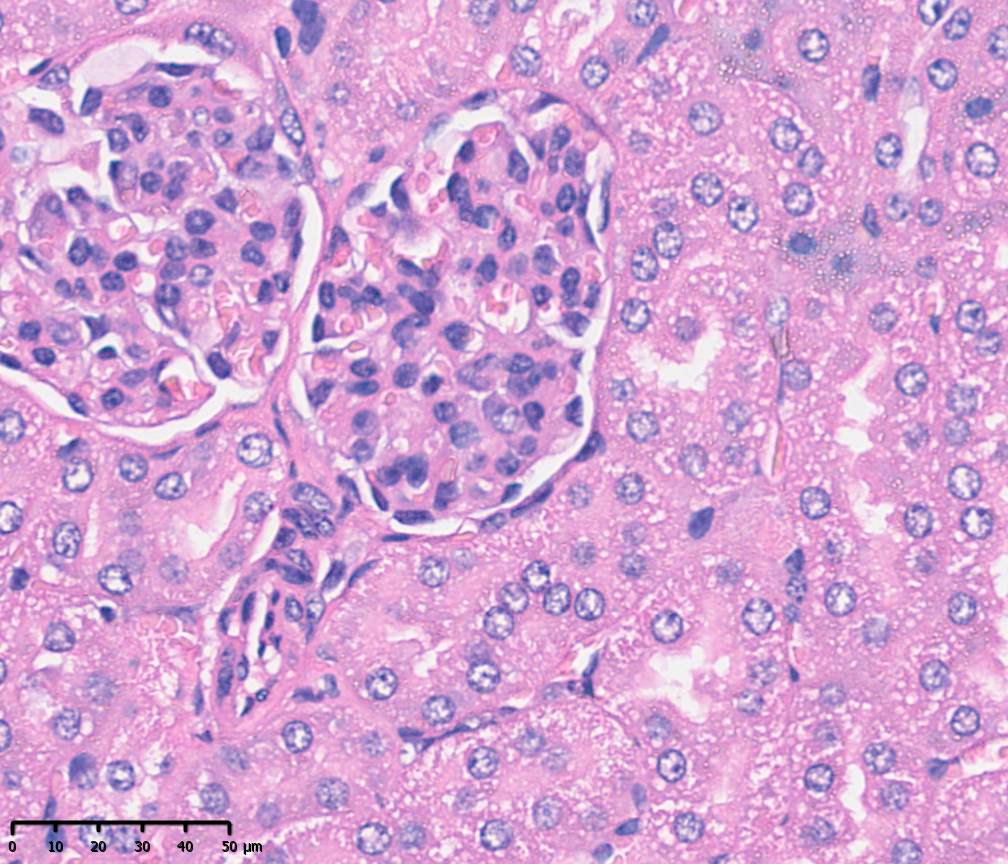

Supplement: Supplementary file 8 — Source data Fig. 7 [file 44319_2024_291_MOESM8_ESM.zip › Figure7G/KO_LPS_KIDNEY.tif]

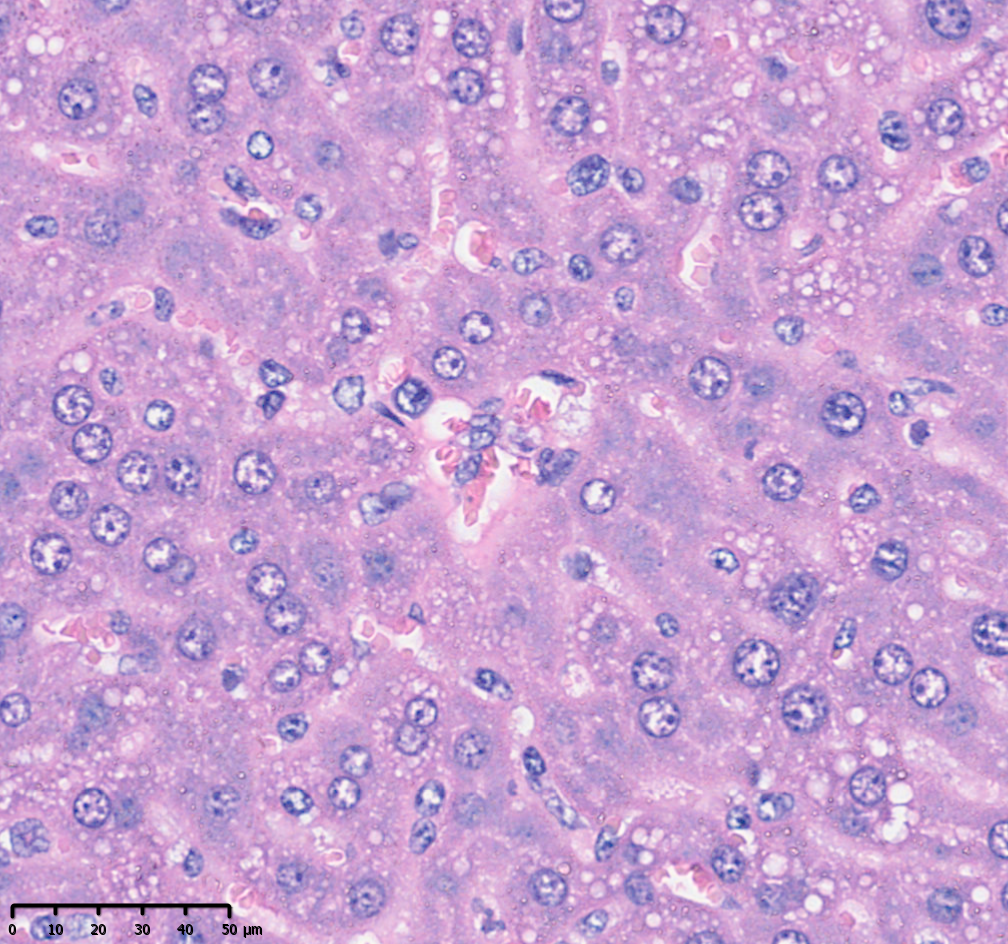

Supplement: Supplementary file 8 — Source data Fig. 7 [file 44319_2024_291_MOESM8_ESM.zip › Figure7G/KO_LPS_LIVER.tif]

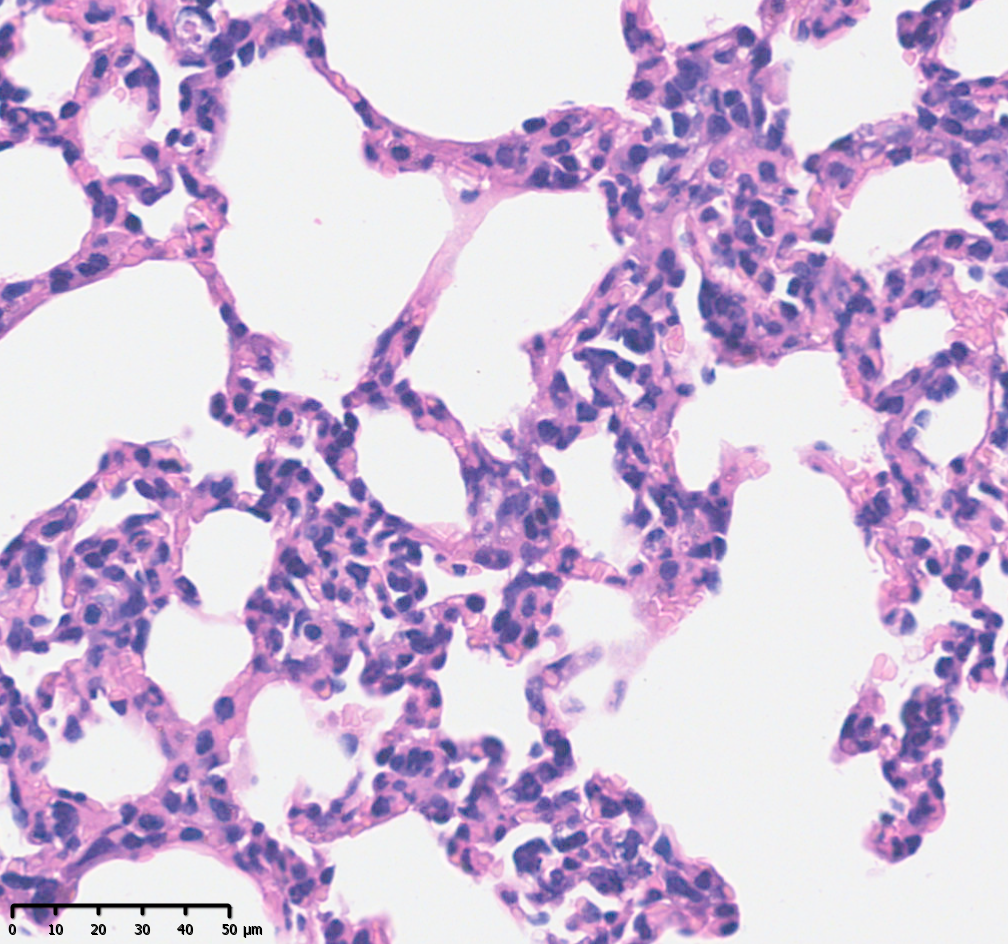

Supplement: Supplementary file 8 — Source data Fig. 7 [file 44319_2024_291_MOESM8_ESM.zip › Figure7G/KO_LPS_LUNG.tif]

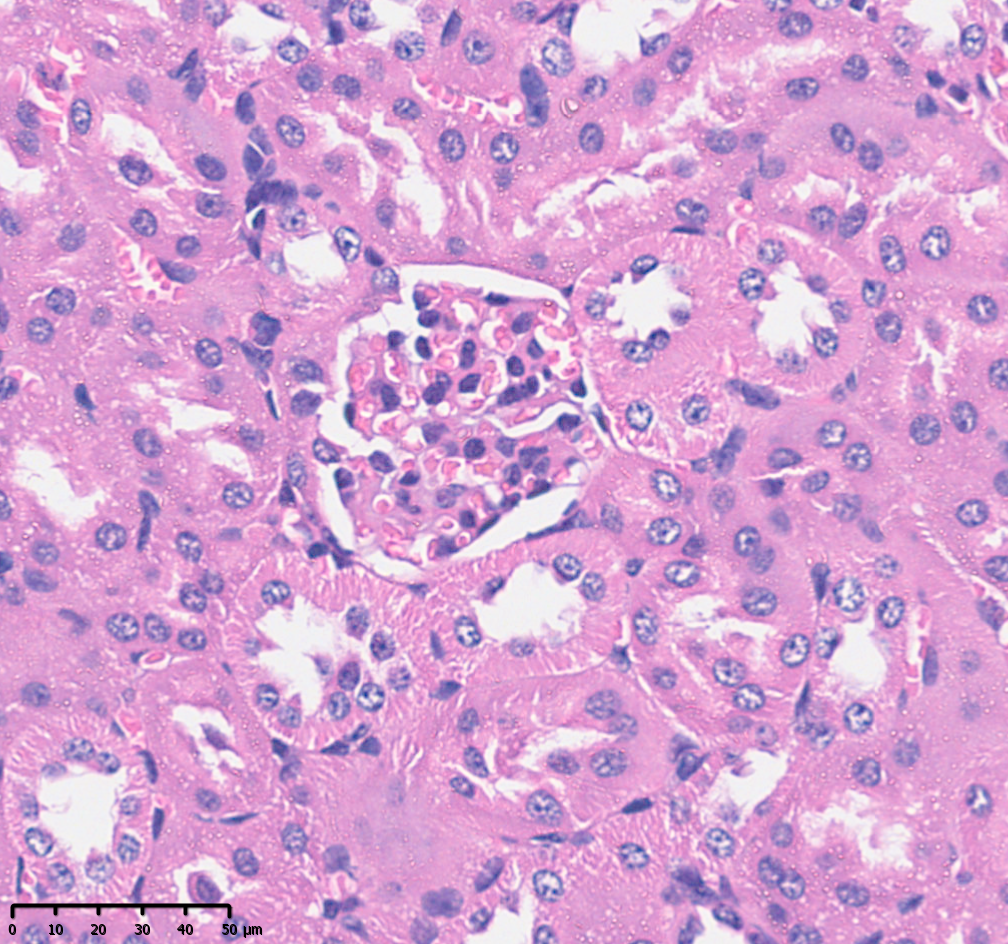

Supplement: Supplementary file 8 — Source data Fig. 7 [file 44319_2024_291_MOESM8_ESM.zip › Figure7G/KO_LPS_MOCK_KIDNEY.tif]

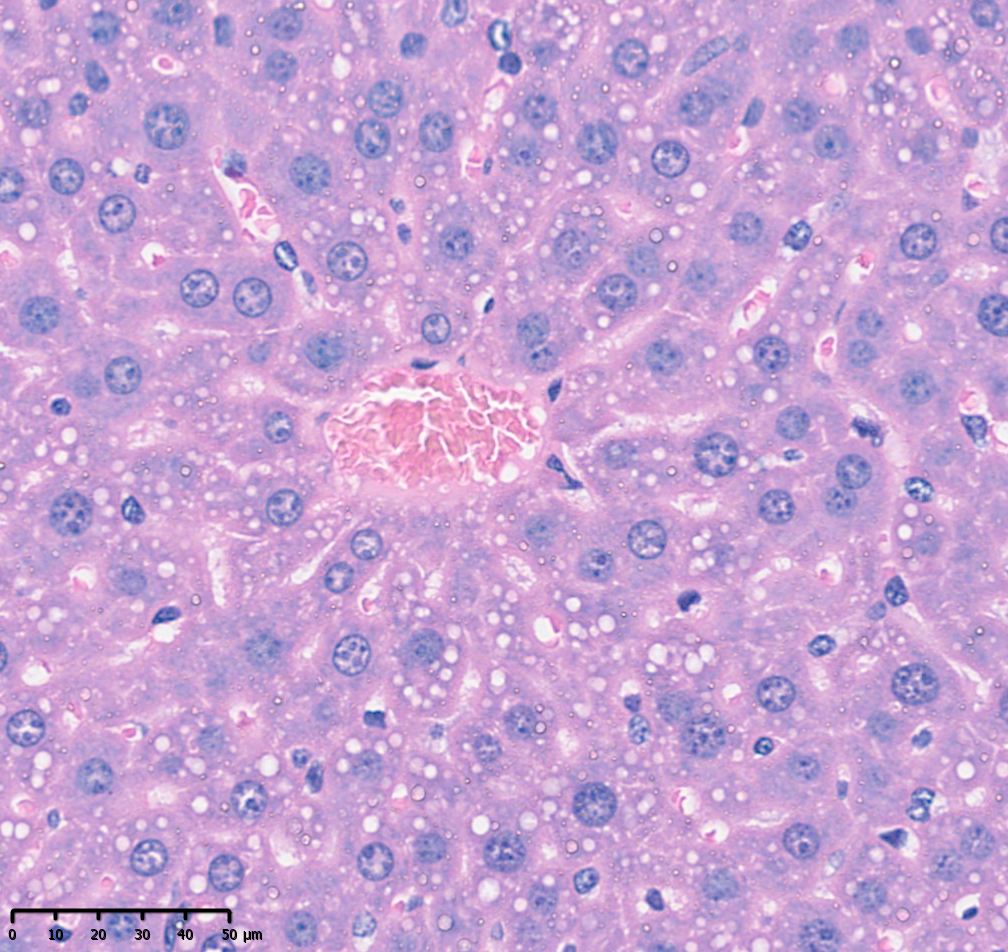

Supplement: Supplementary file 8 — Source data Fig. 7 [file 44319_2024_291_MOESM8_ESM.zip › Figure7G/KO_LPS_MOCK_LIVER.tif]

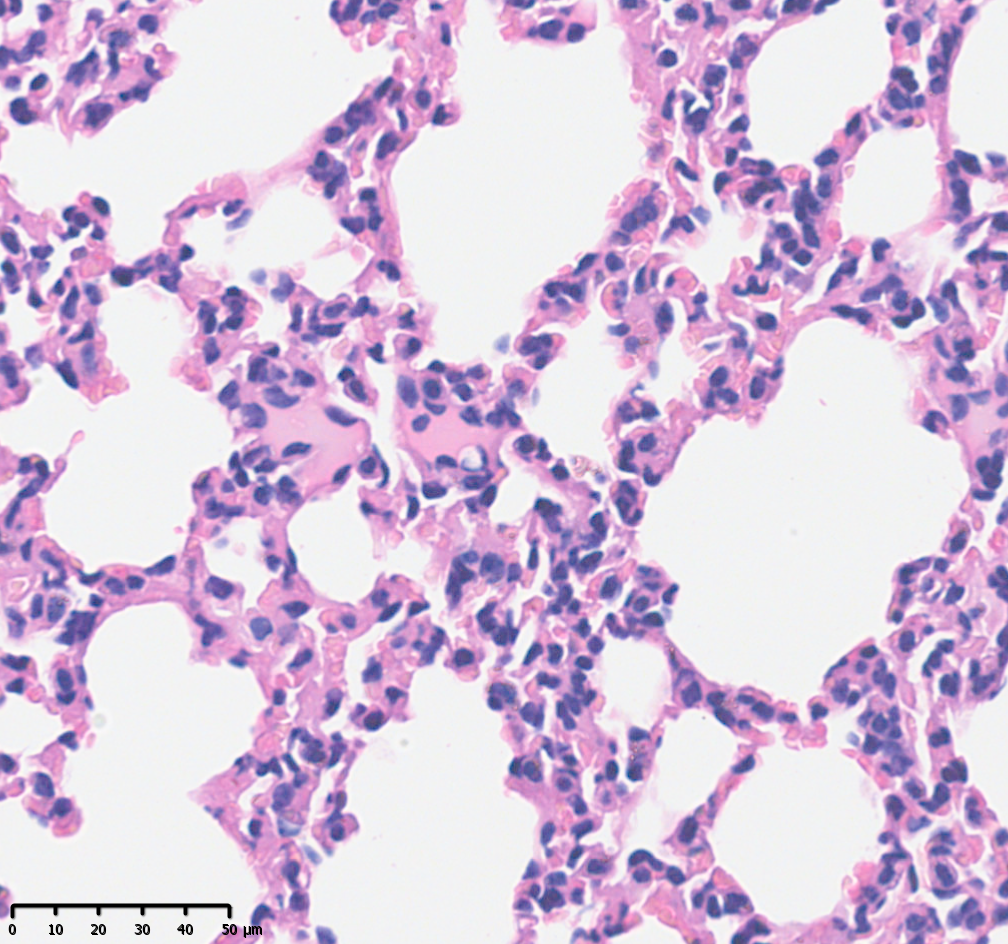

Supplement: Supplementary file 8 — Source data Fig. 7 [file 44319_2024_291_MOESM8_ESM.zip › Figure7G/KO_LPS_MOCK_LUNG.tif]

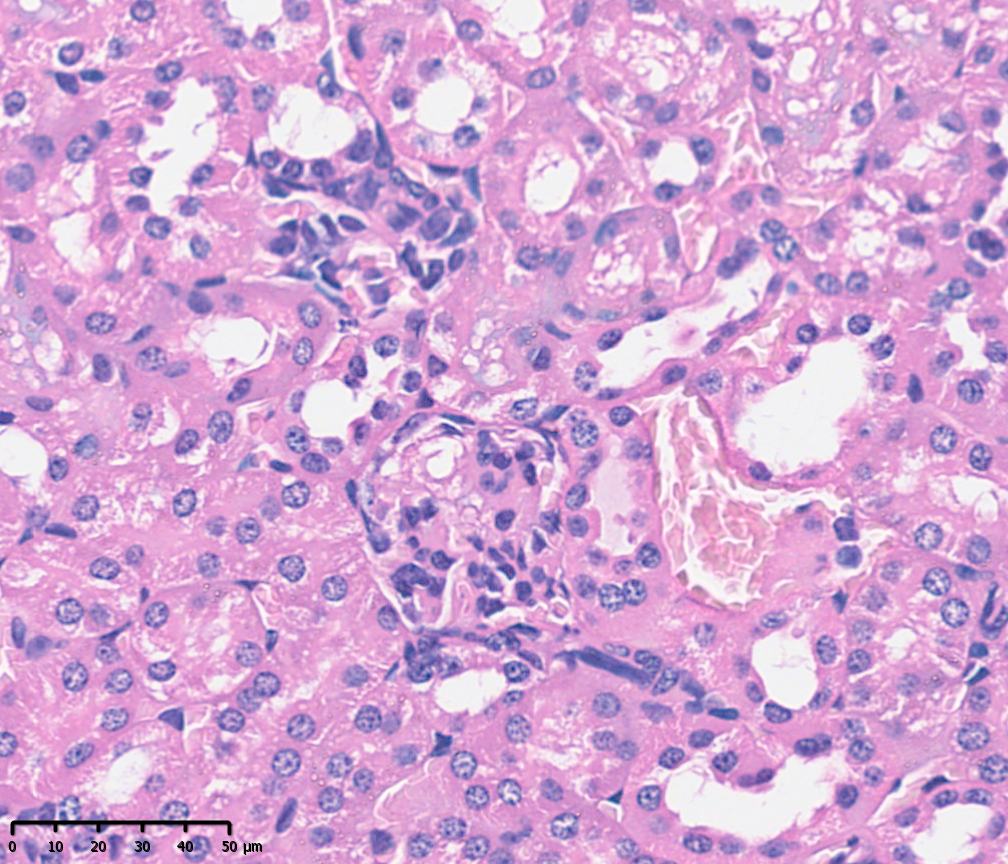

Supplement: Supplementary file 8 — Source data Fig. 7 [file 44319_2024_291_MOESM8_ESM.zip › Figure7G/KO_LPS_TLR4_KIDNEY.tif]

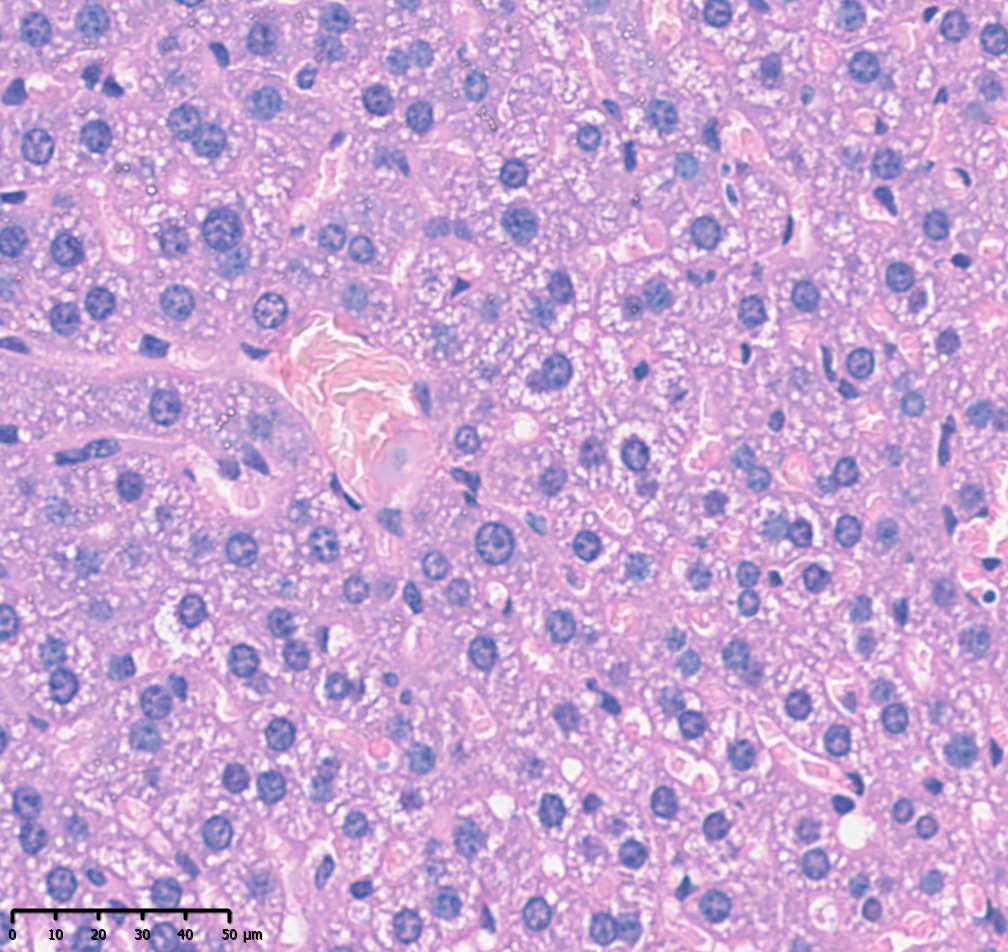

Supplement: Supplementary file 8 — Source data Fig. 7 [file 44319_2024_291_MOESM8_ESM.zip › Figure7G/KO_LPS_TLR4_LIVER.tif]

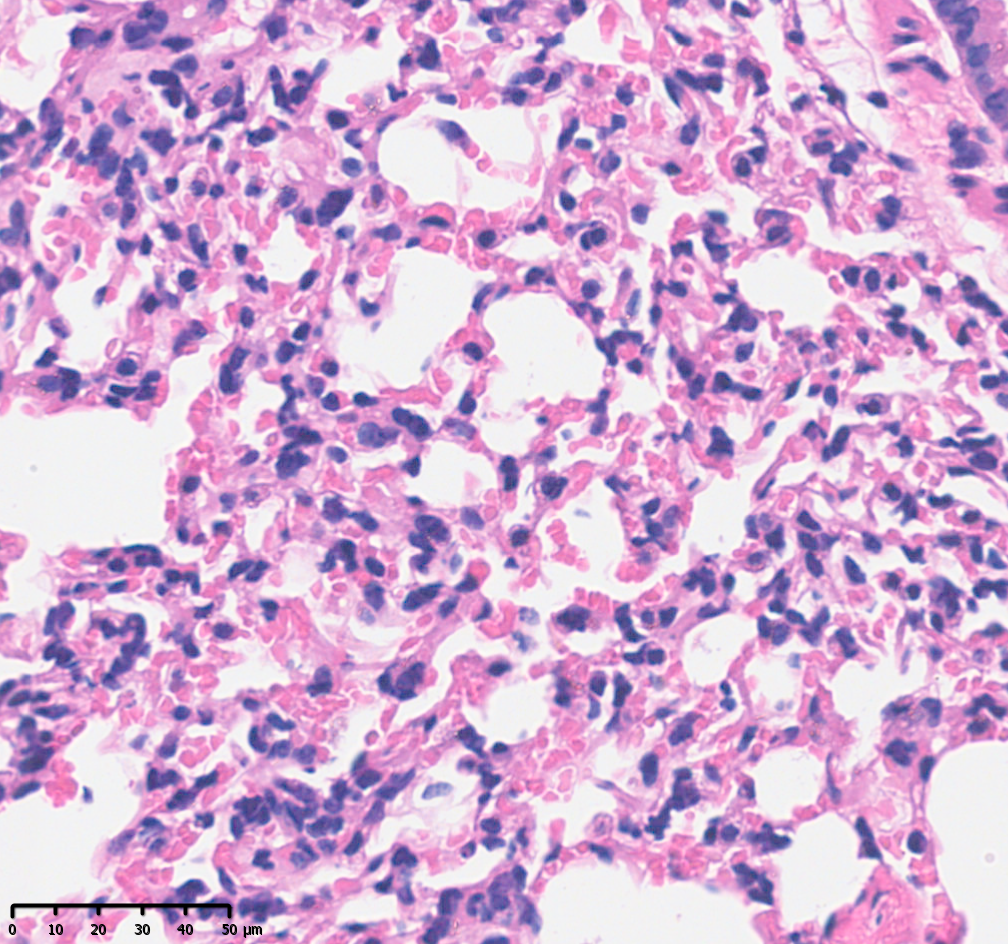

Supplement: Supplementary file 8 — Source data Fig. 7 [file 44319_2024_291_MOESM8_ESM.zip › Figure7G/KO_LPS_TLR4_LUNG.tif]

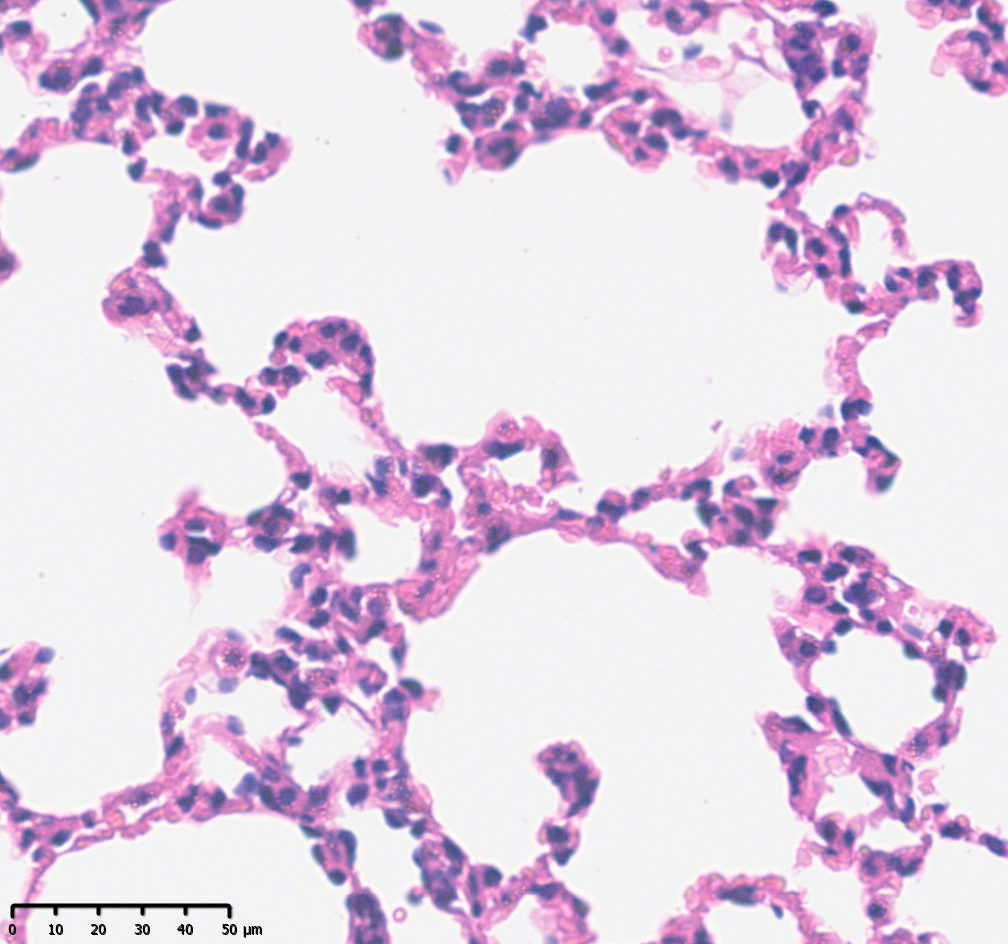

Supplement: Supplementary file 8 — Source data Fig. 7 [file 44319_2024_291_MOESM8_ESM.zip › Figure7G/KO_LUNG.tif]

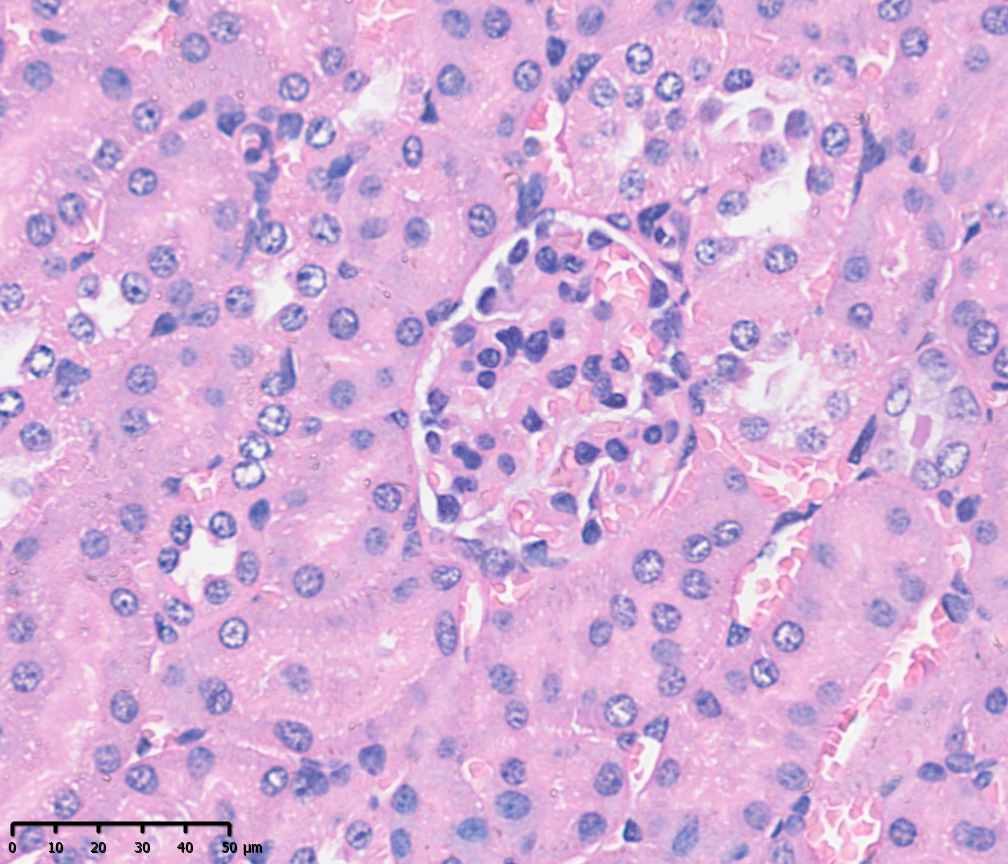

Supplement: Supplementary file 8 — Source data Fig. 7 [file 44319_2024_291_MOESM8_ESM.zip › Figure7G/WT-KIDNEY.tif]

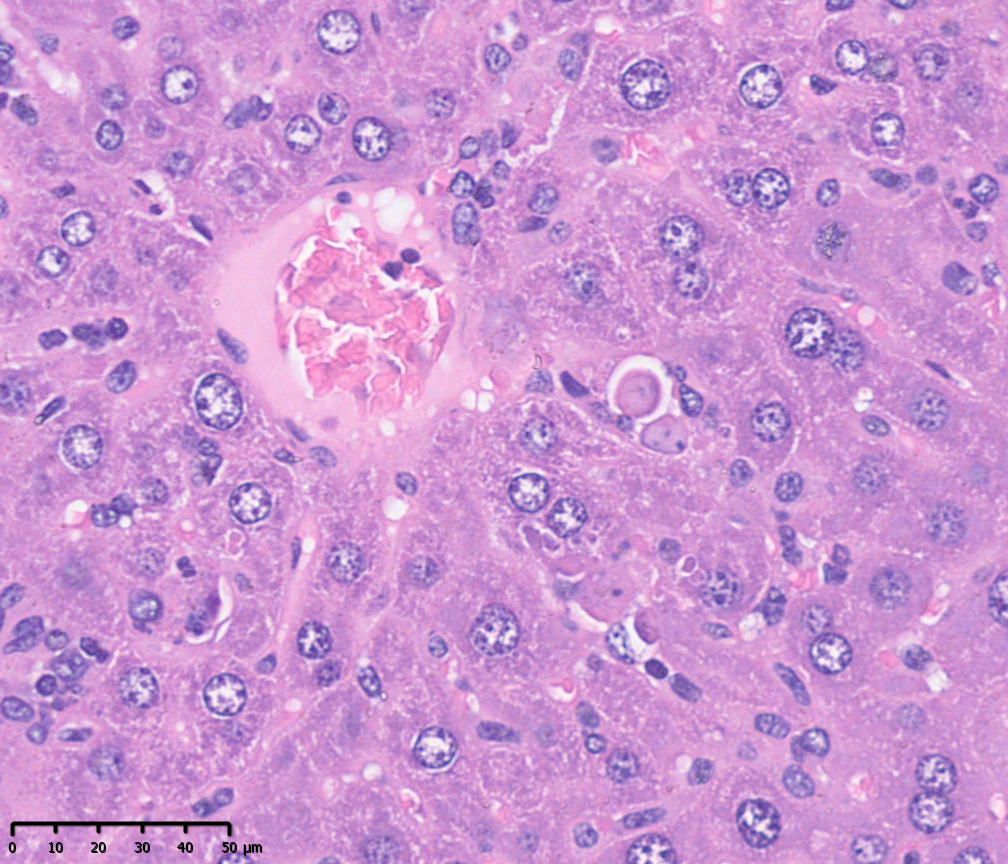

Supplement: Supplementary file 8 — Source data Fig. 7 [file 44319_2024_291_MOESM8_ESM.zip › Figure7G/WT-LIVER.tif]

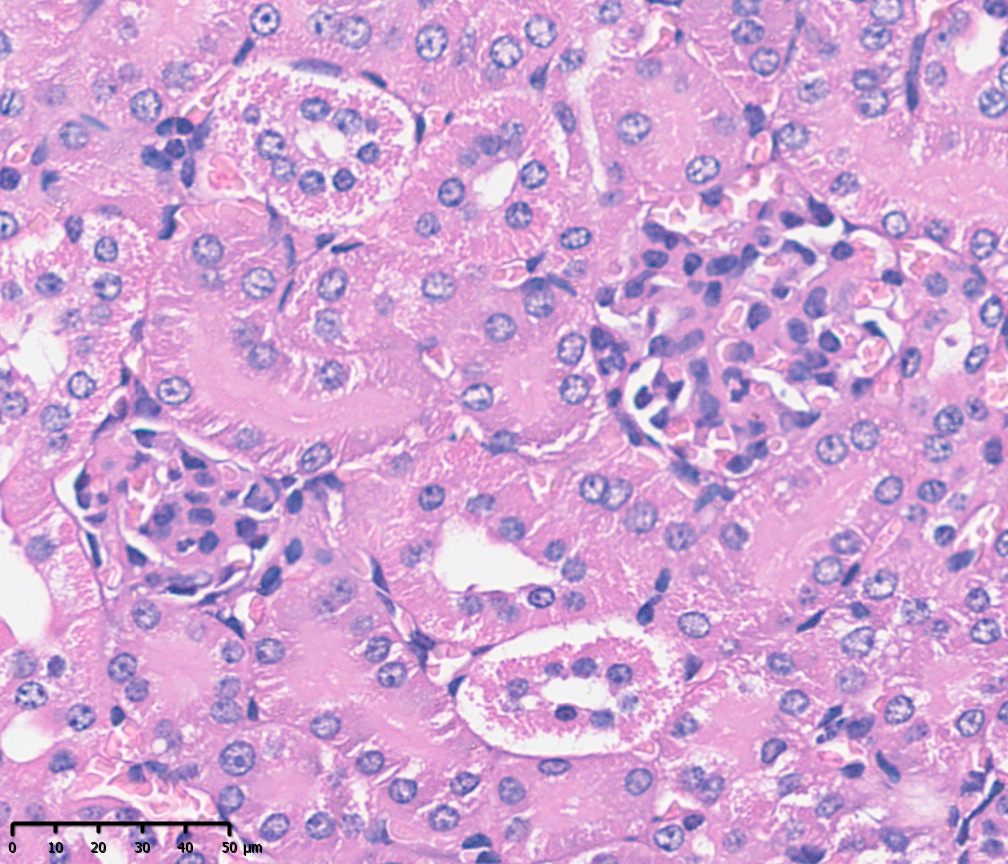

Supplement: Supplementary file 8 — Source data Fig. 7 [file 44319_2024_291_MOESM8_ESM.zip › Figure7G/WT_LPS_KIDNEY.tif]

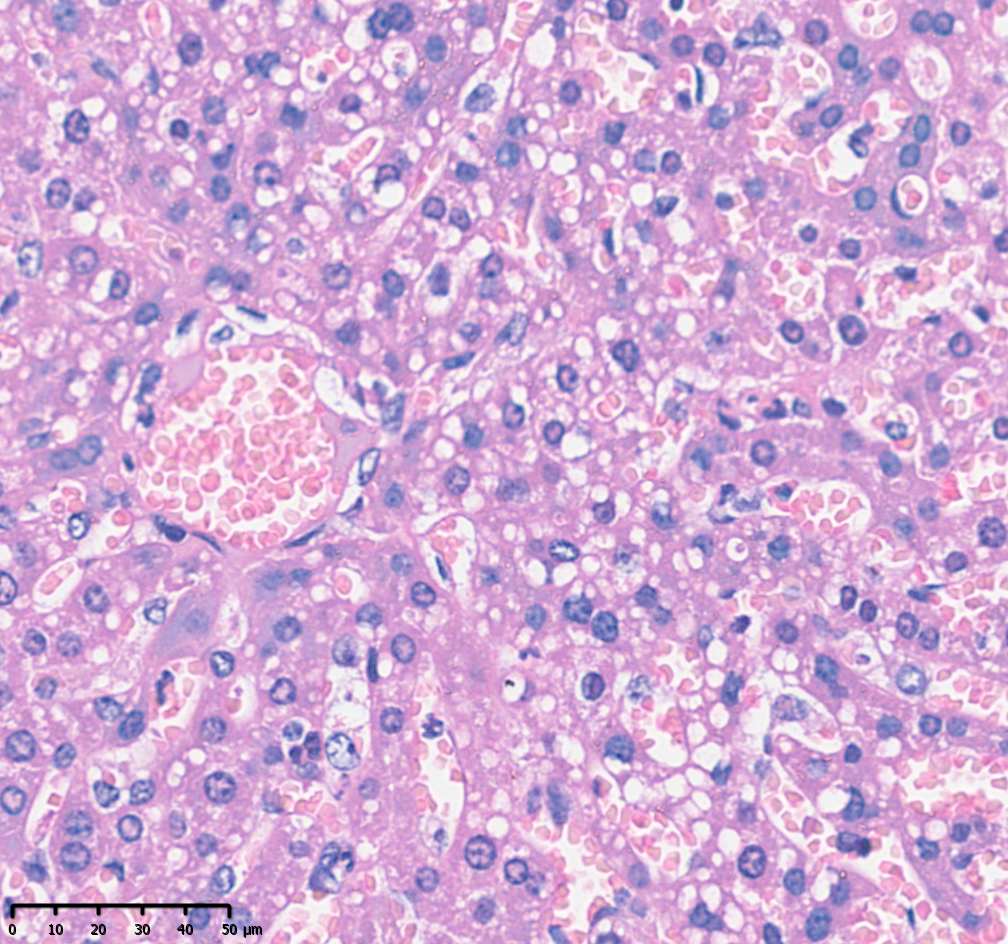

Supplement: Supplementary file 8 — Source data Fig. 7 [file 44319_2024_291_MOESM8_ESM.zip › Figure7G/WT_LPS_LIVER.tif]

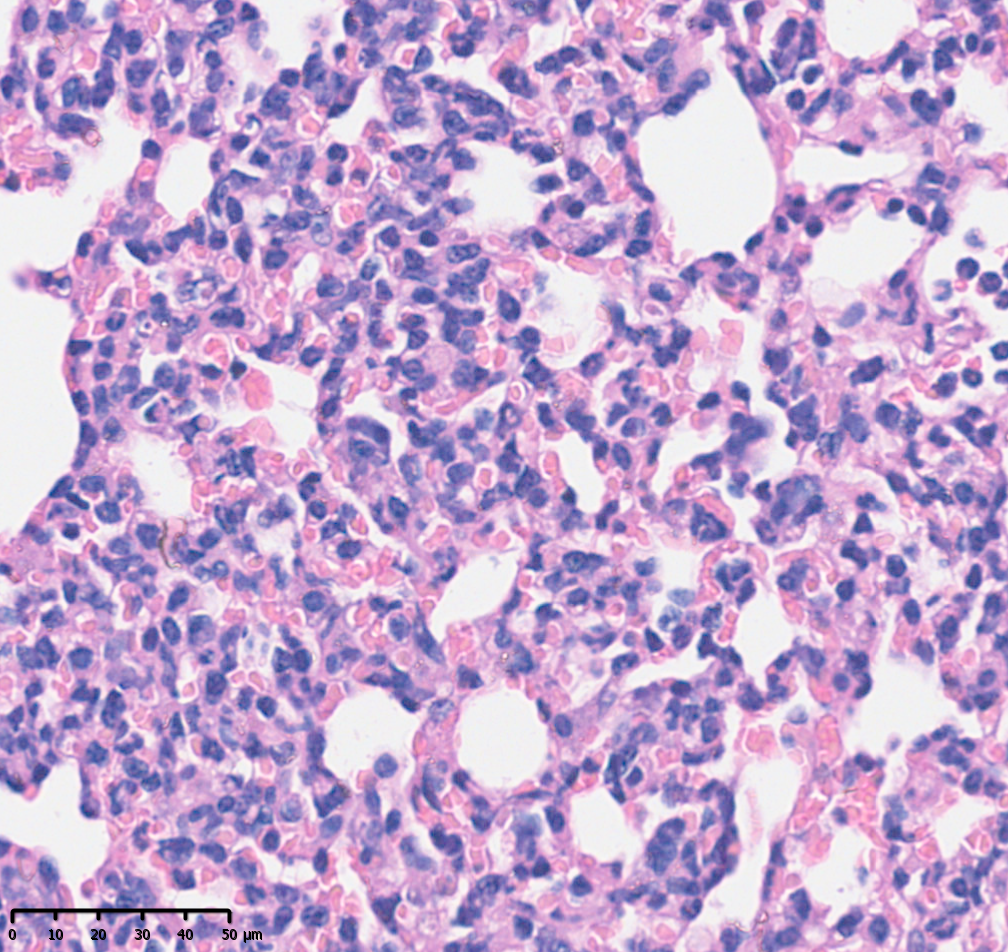

Supplement: Supplementary file 8 — Source data Fig. 7 [file 44319_2024_291_MOESM8_ESM.zip › Figure7G/WT_LPS_LUNG.tif]

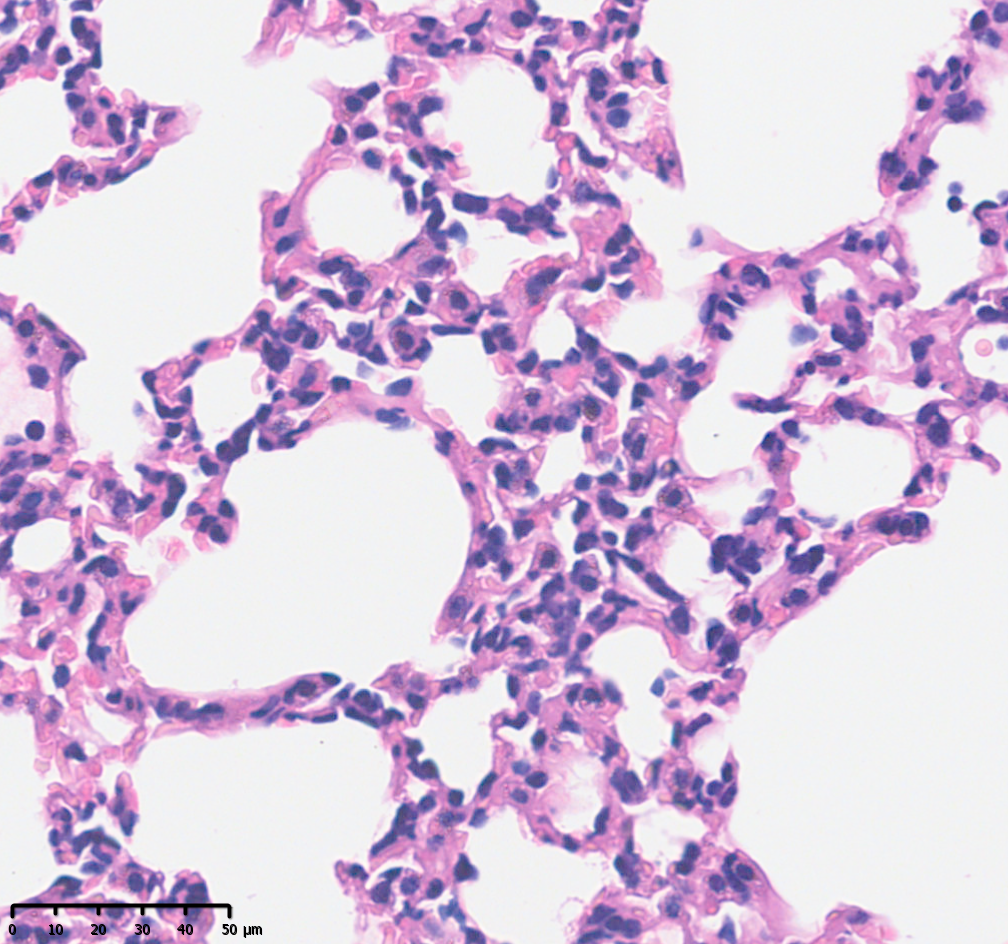

Supplement: Supplementary file 8 — Source data Fig. 7 [file 44319_2024_291_MOESM8_ESM.zip › Figure7G/WT_LUNG.tif]

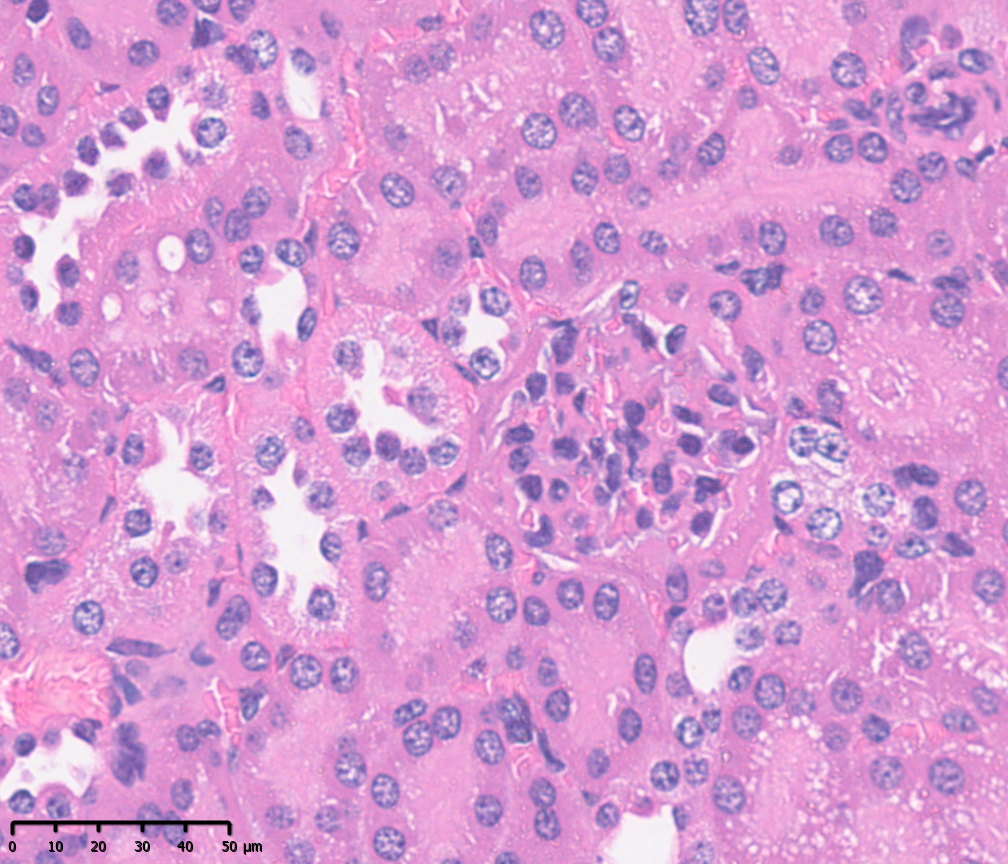

Supplement: Supplementary file 9 — Source data Fig. 8 [file 44319_2024_291_MOESM9_ESM.zip › Figure8G/KO_CLP_KIDNEY.tif]

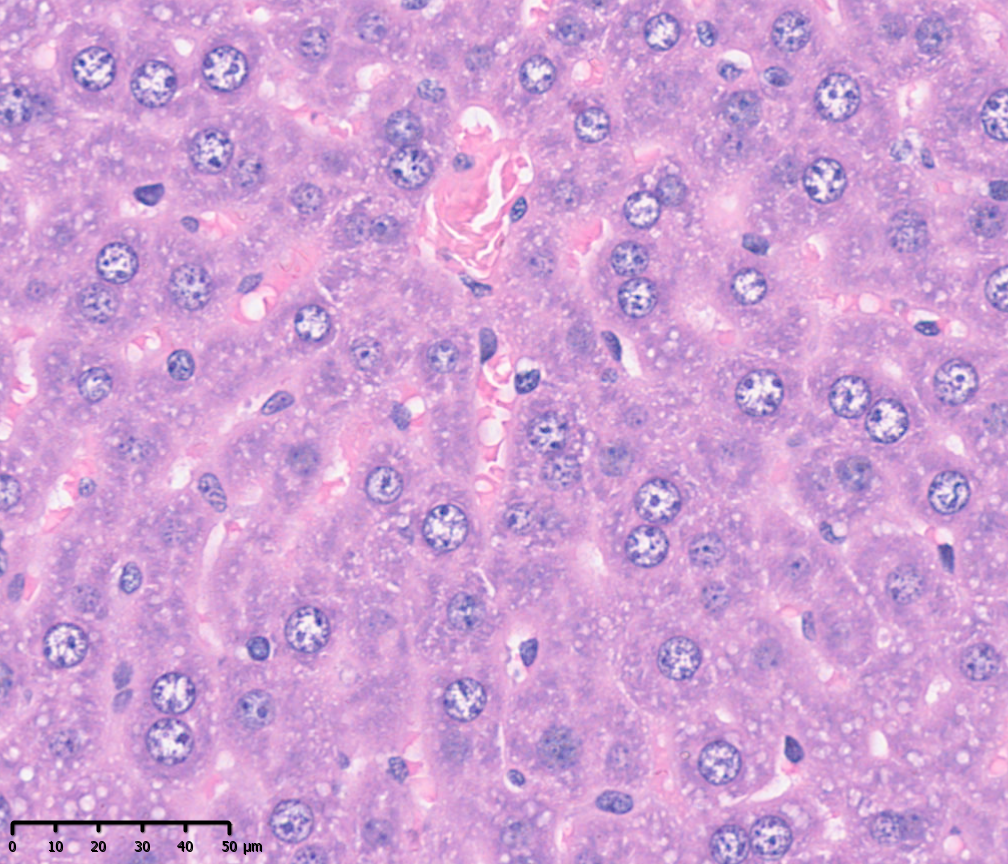

Supplement: Supplementary file 9 — Source data Fig. 8 [file 44319_2024_291_MOESM9_ESM.zip › Figure8G/KO_CLP_LIVER.tif]

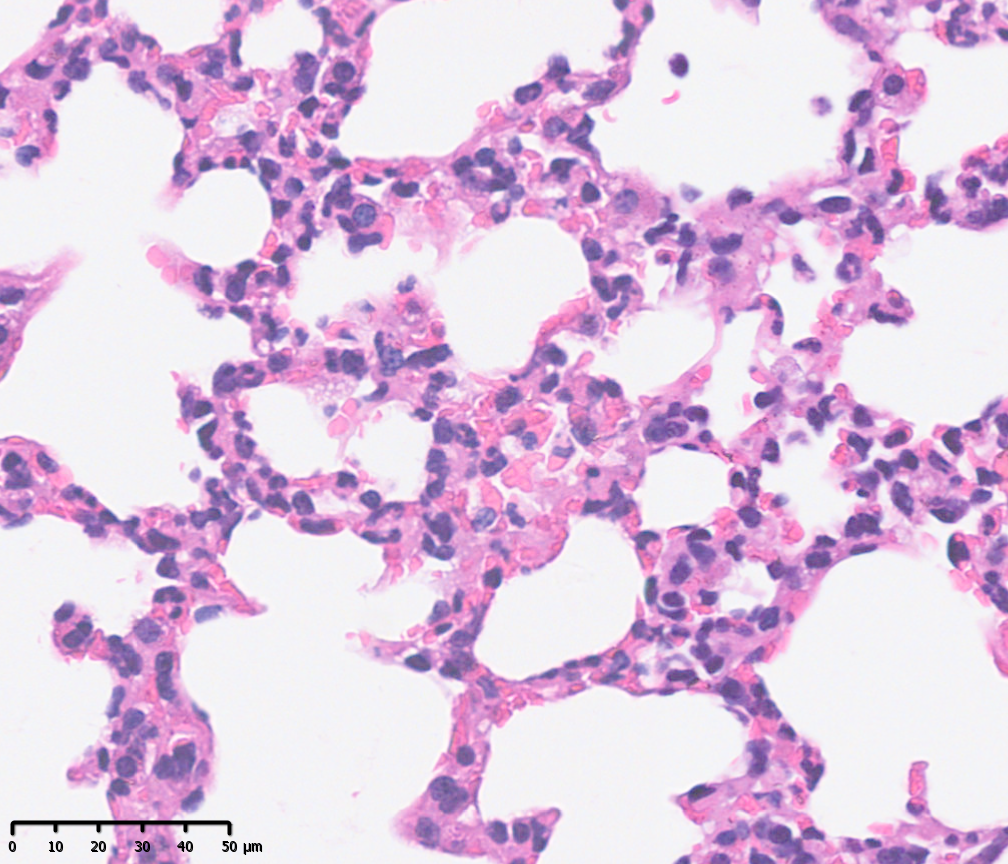

Supplement: Supplementary file 9 — Source data Fig. 8 [file 44319_2024_291_MOESM9_ESM.zip › Figure8G/KO_CLP_LUNG.tif]

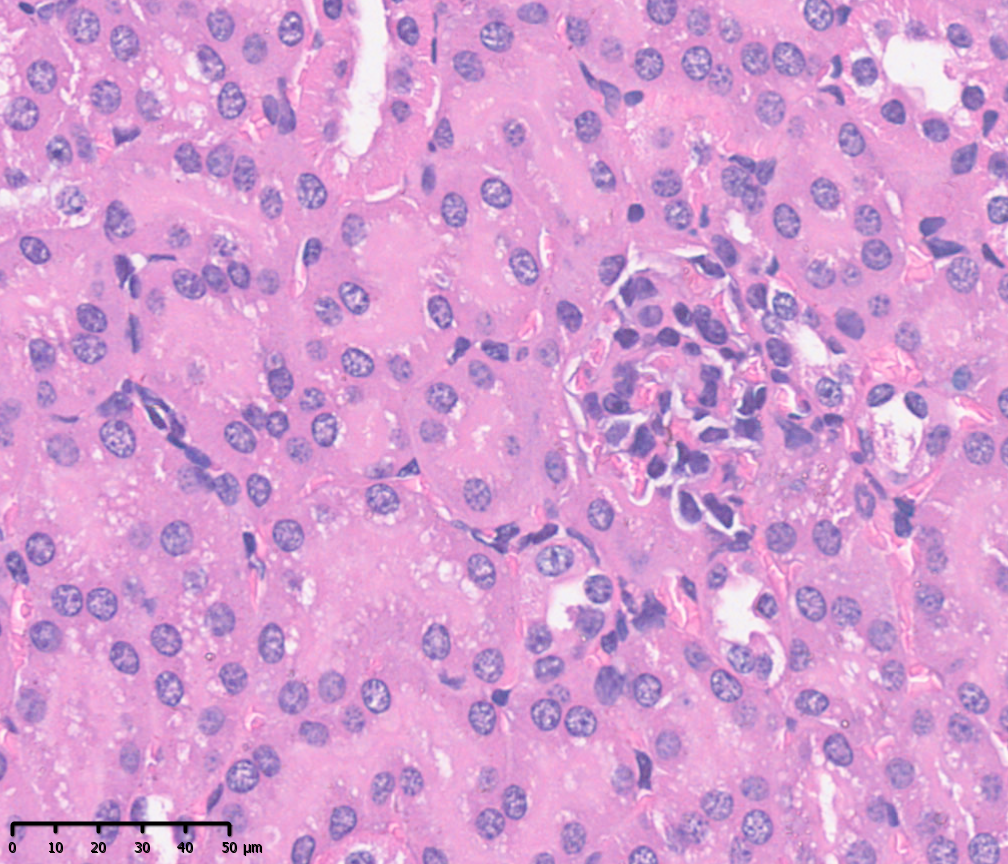

Supplement: Supplementary file 9 — Source data Fig. 8 [file 44319_2024_291_MOESM9_ESM.zip › Figure8G/KO_CLP_MOCK_KIDNEY.tif]

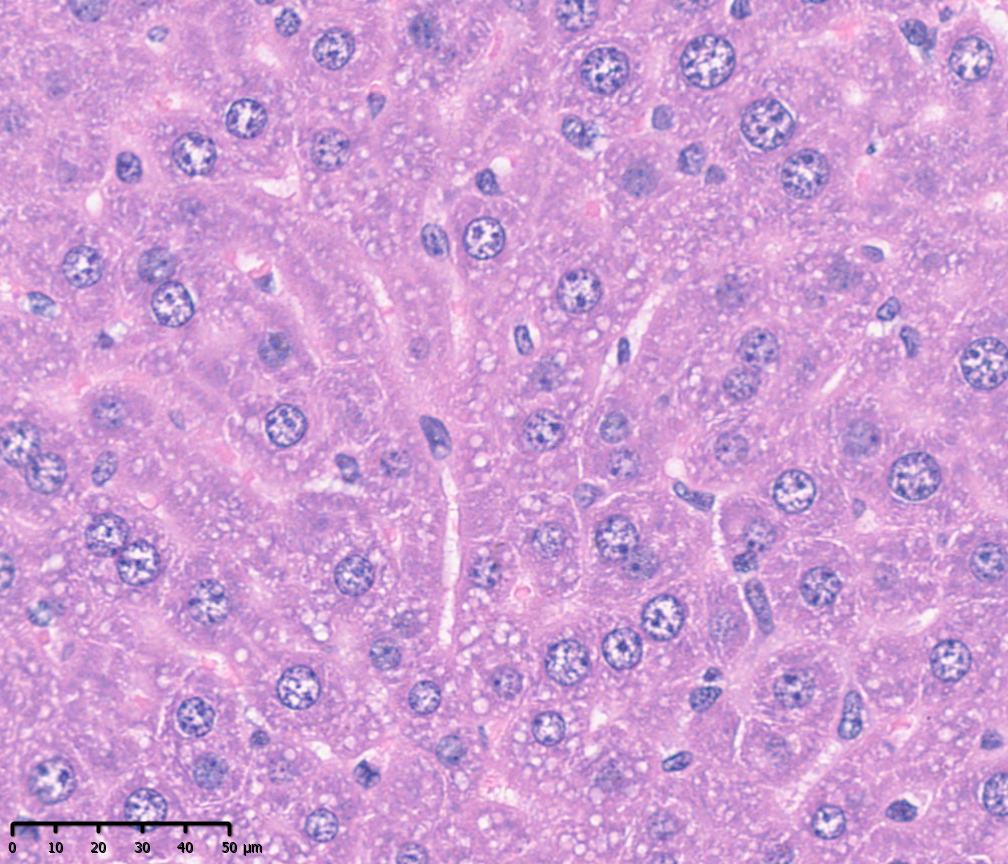

Supplement: Supplementary file 9 — Source data Fig. 8 [file 44319_2024_291_MOESM9_ESM.zip › Figure8G/KO_CLP_MOCK_LIVER.tif]

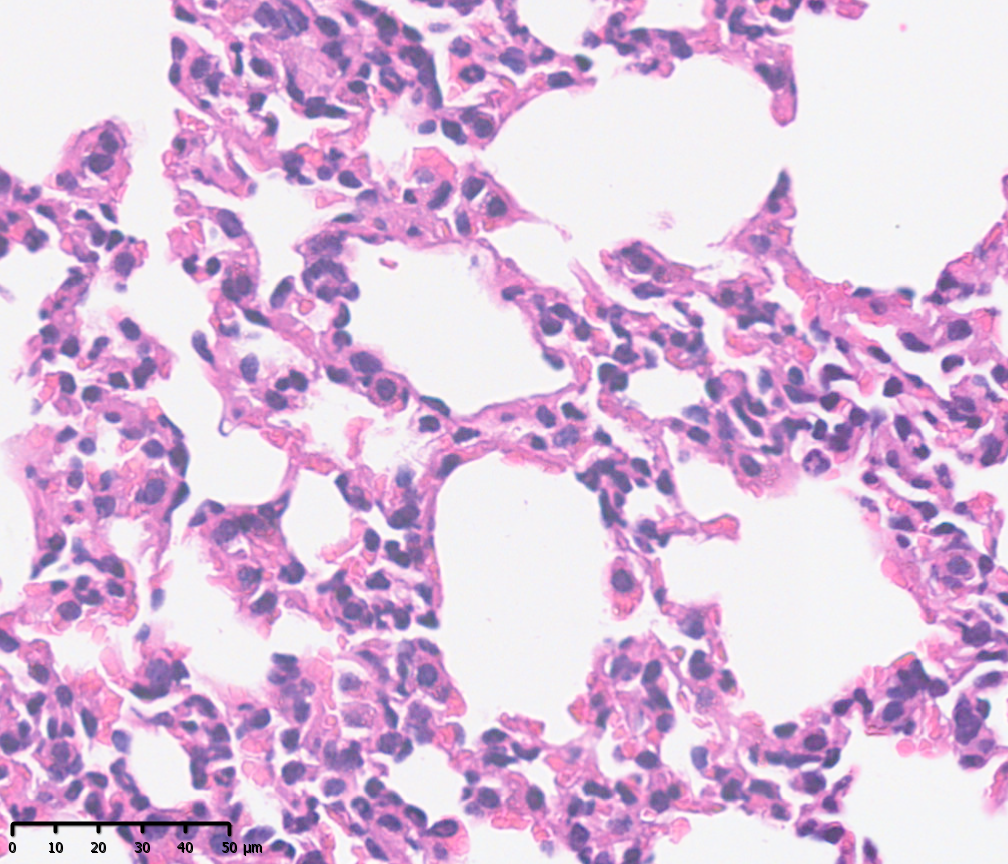

Supplement: Supplementary file 9 — Source data Fig. 8 [file 44319_2024_291_MOESM9_ESM.zip › Figure8G/KO_CLP_MOCK_LUNG.tif]

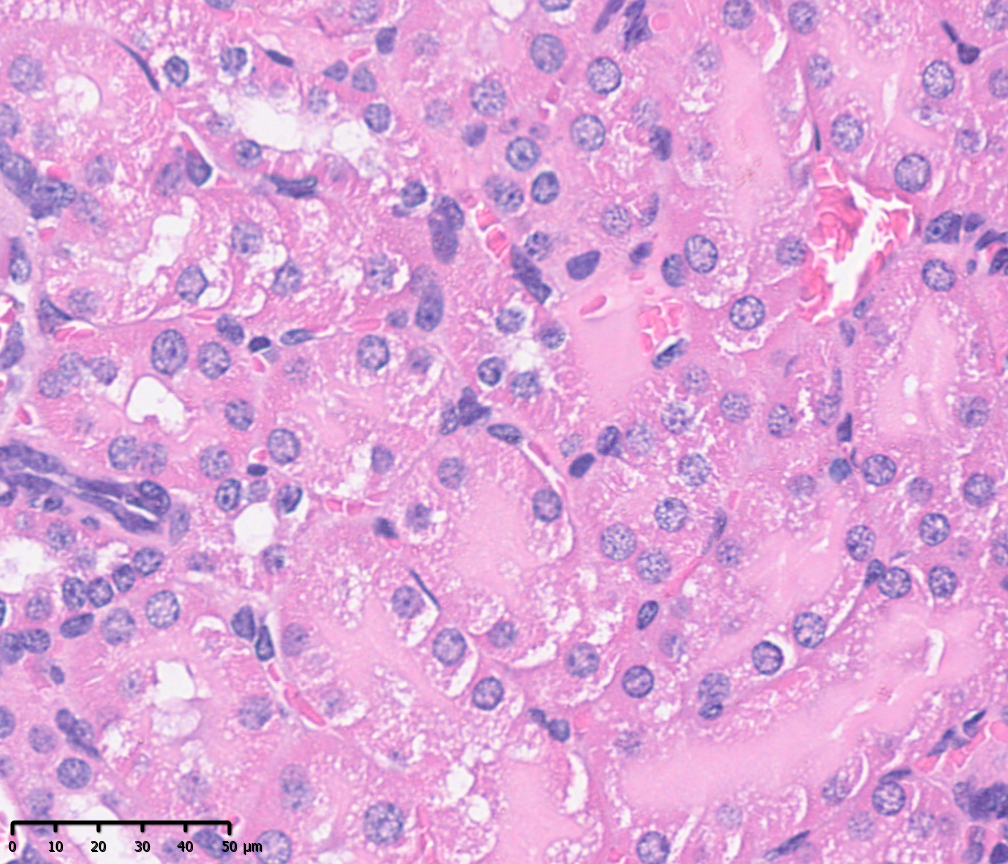

Supplement: Supplementary file 9 — Source data Fig. 8 [file 44319_2024_291_MOESM9_ESM.zip › Figure8G/KO_CLP_TLR4_KIDNEY.tif]

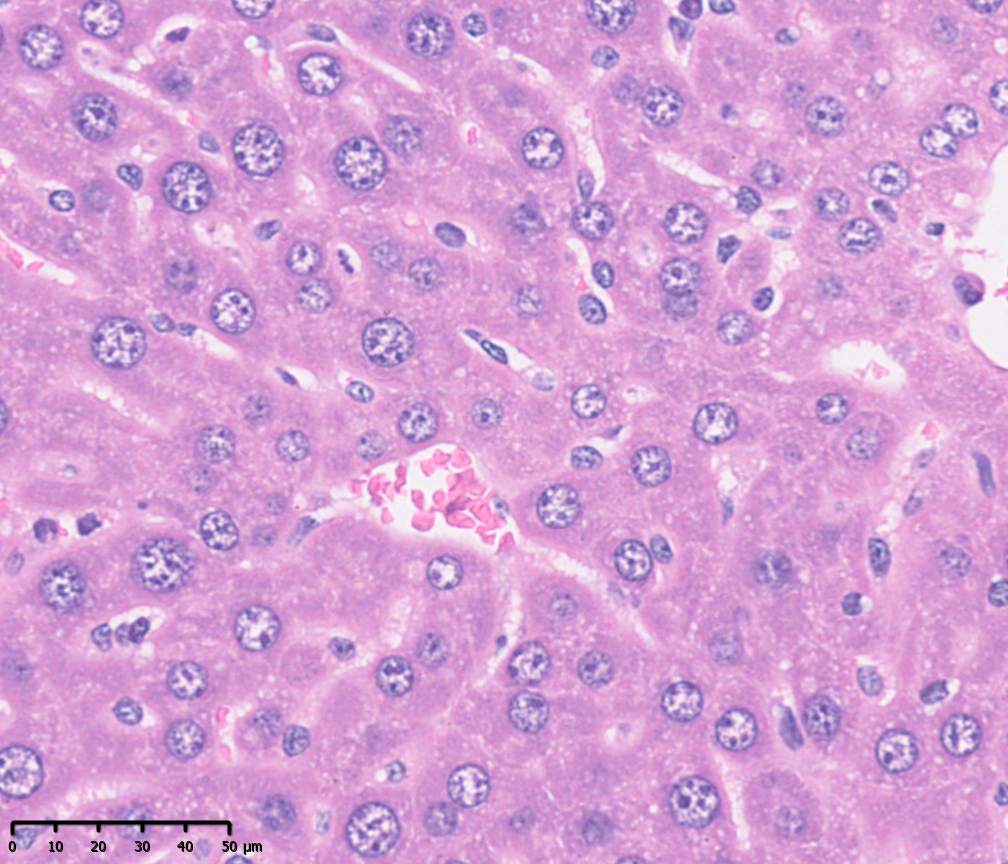

Supplement: Supplementary file 9 — Source data Fig. 8 [file 44319_2024_291_MOESM9_ESM.zip › Figure8G/KO_CLP_TLR4_LIVER.tif]

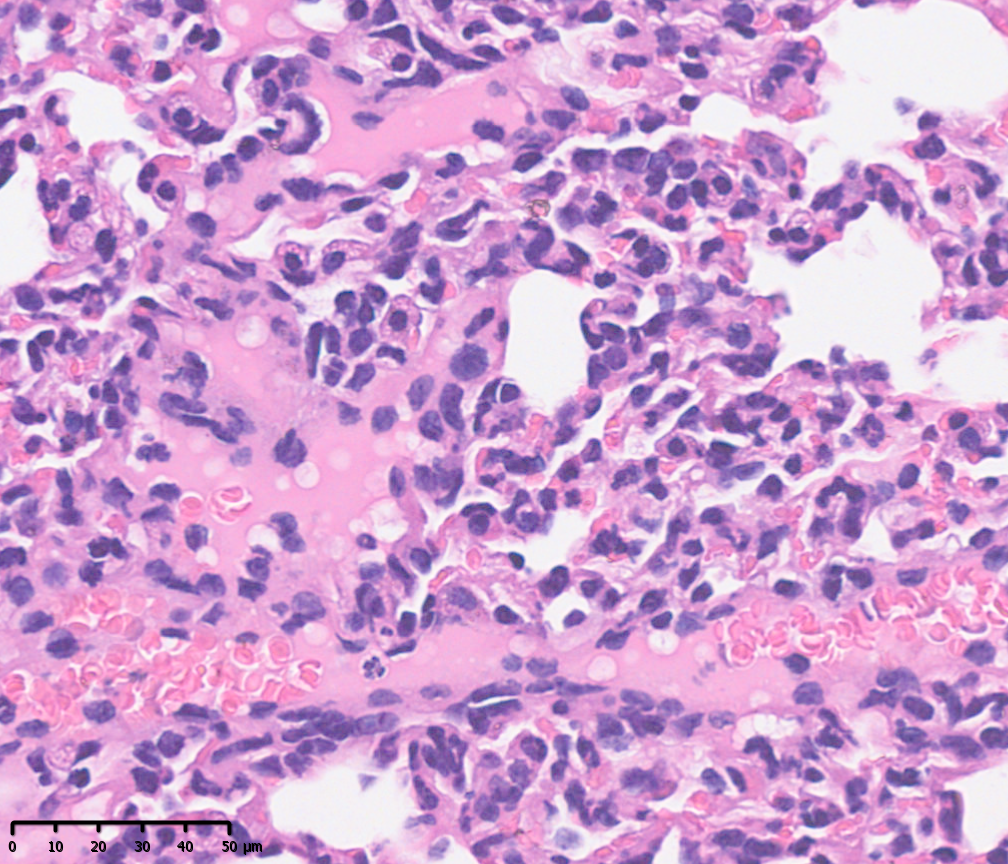

Supplement: Supplementary file 9 — Source data Fig. 8 [file 44319_2024_291_MOESM9_ESM.zip › Figure8G/KO_CLP_TLR4_LUNG.tif]

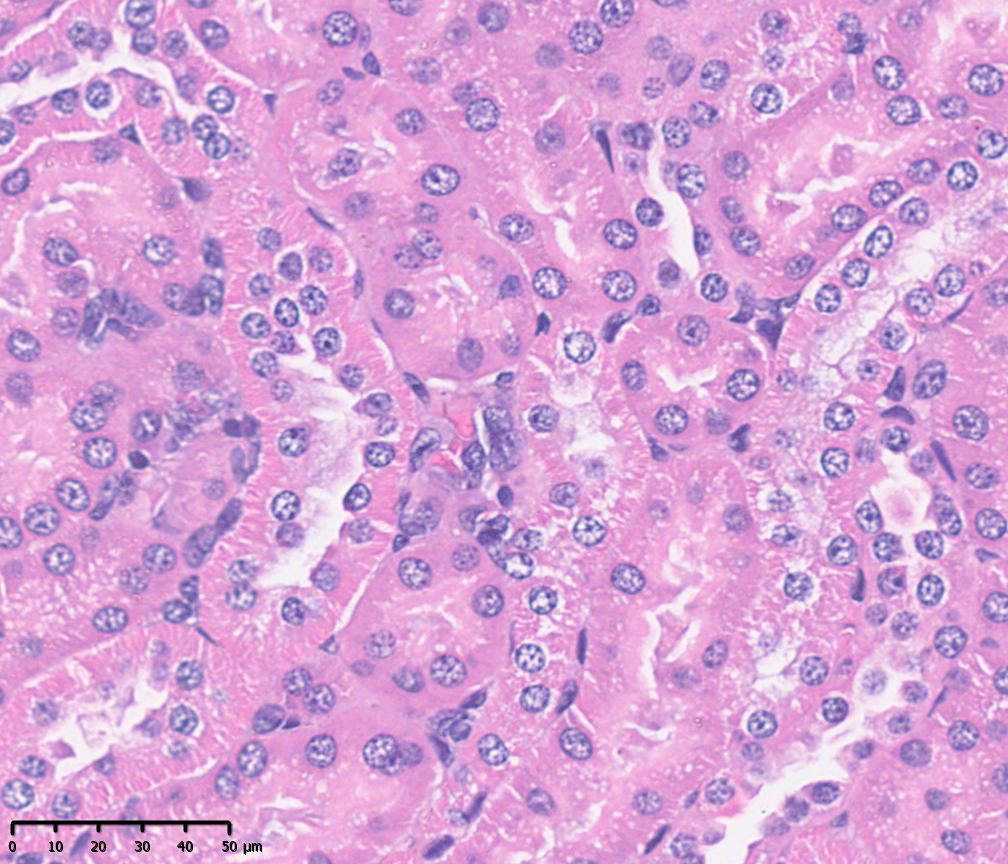

Supplement: Supplementary file 9 — Source data Fig. 8 [file 44319_2024_291_MOESM9_ESM.zip › Figure8G/WT_CLP_KIDNEY.tif]

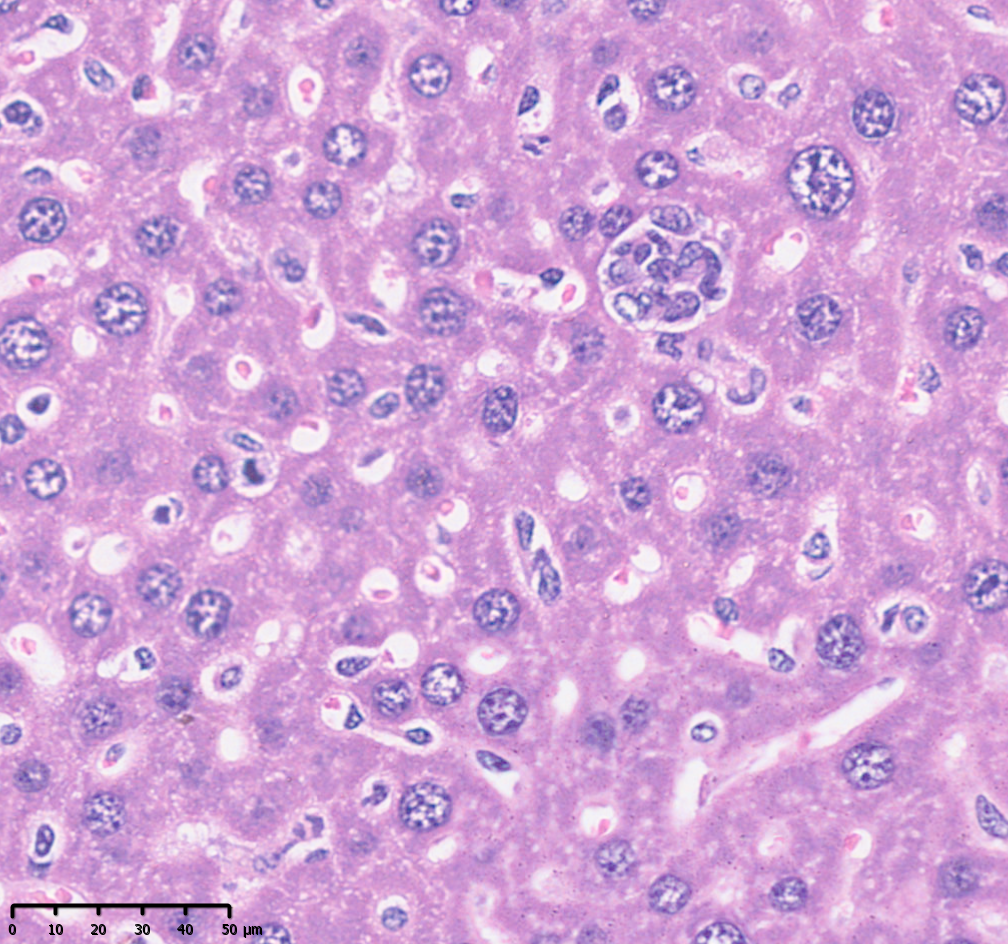

Supplement: Supplementary file 9 — Source data Fig. 8 [file 44319_2024_291_MOESM9_ESM.zip › Figure8G/WT_CLP_LIVER.tif]

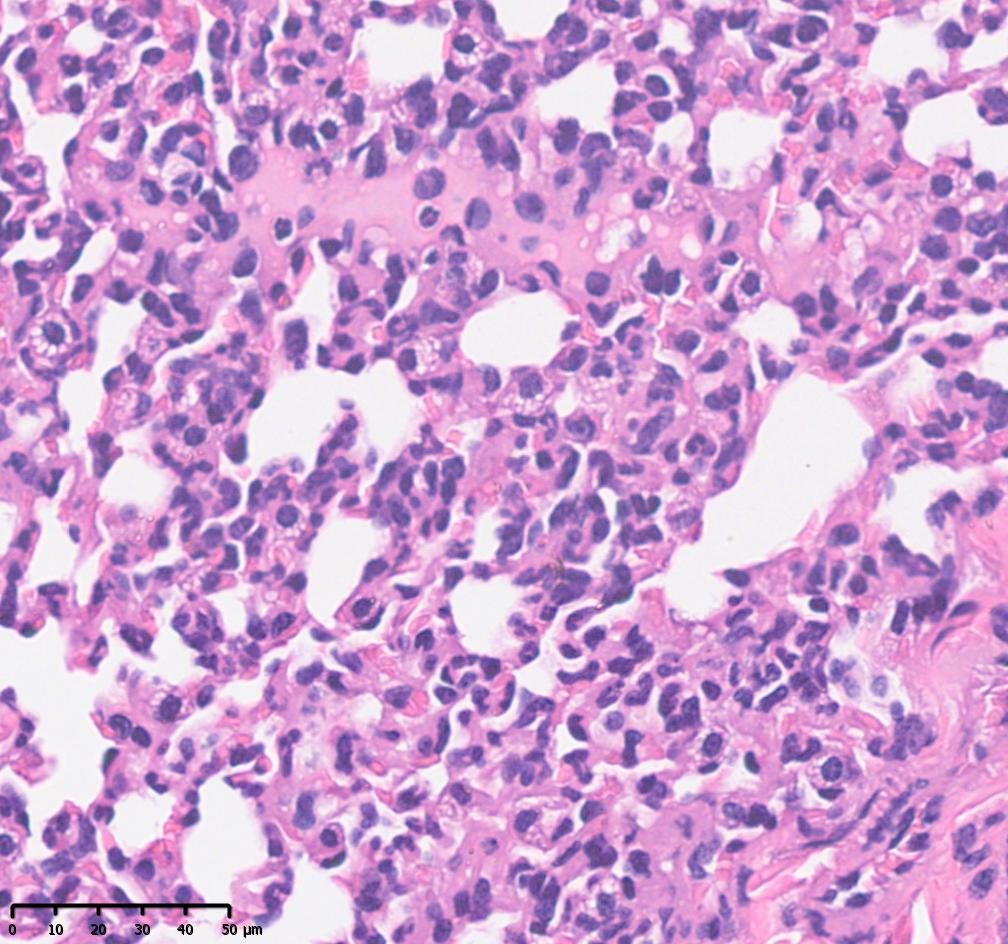

Supplement: Supplementary file 9 — Source data Fig. 8 [file 44319_2024_291_MOESM9_ESM.zip › Figure8G/WT_CLP_LUNG.tif]
