# Supplementary material for: CMTM3 regulates neutrophil activation and aggravates sepsis through TLR4 signaling
Source: EMBO Rep. 2024 Oct 25;25(12):5456–77. doi: 10.1038/s44319-024-00291-7 (PMC11624275; doi:10.1038/s44319-024-00291-7)
Supplement: Supplementary file 10 — Expanded View Figures [file 44319_2024_291_MOESM10_ESM.pdf]

Expanded View Figures

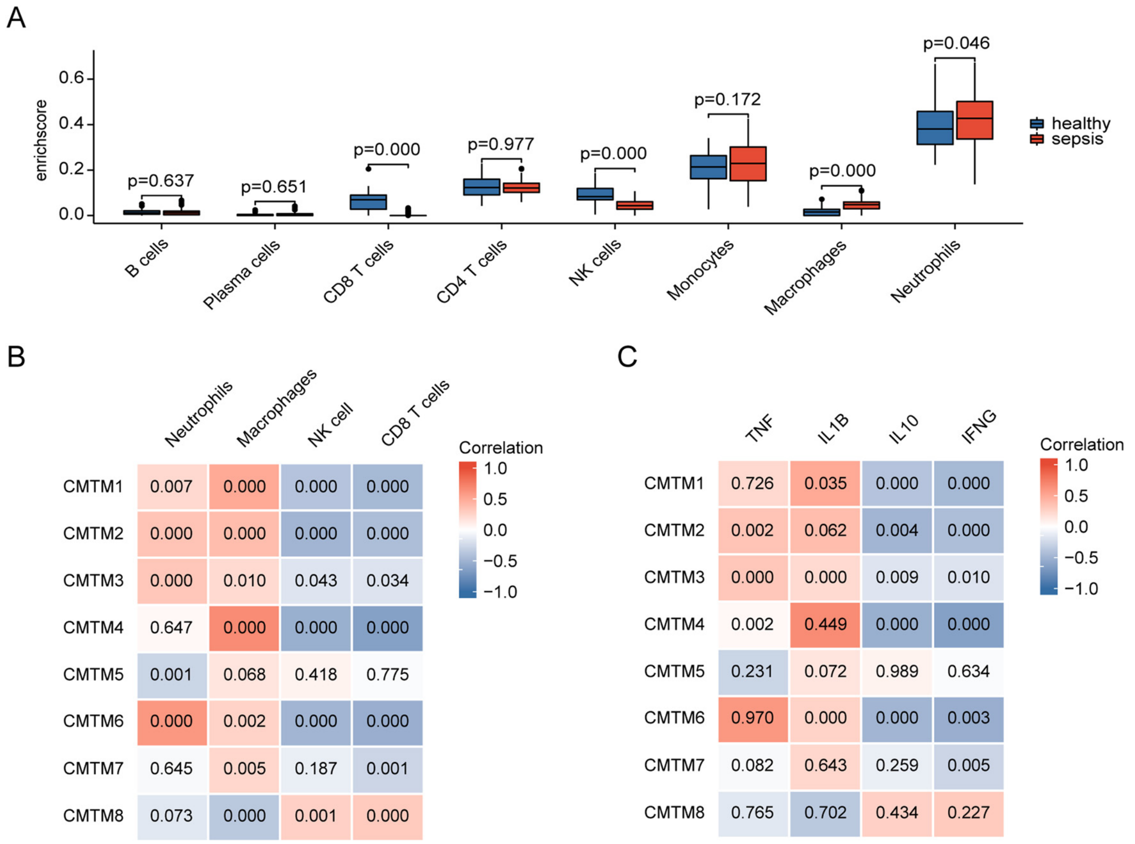

Figure EV1. Expression and correlation analysis of *CMTM* family in sepsis using public datasets.

(**A**) Differences in immune cell abundance between sepsis and healthy groups (healthy = 40, sepsis = 106). (**B**) Correlation analysis between *CMTM* family members and immune cells ($n = 146$). (**C**) Correlation analysis between *CMTM* family members and inflammatory cytokines ($n = 146$). Data information: In (**A**), the boxplot divides the data into quartiles, with the lower and upper edges of the box typically representing the first quartile (Q1) and the third quartile (Q3), respectively. The horizontal line inside the box represents the median, and the whiskers extend to the minimum and maximum values (outliers are indicated by dots, which is the median ± 2 times the interquartile range). In (**B**, **C**), the exact *p*-values are provided in the heatmap, with colors representing the range of correlation coefficients.

 © The Author(s)

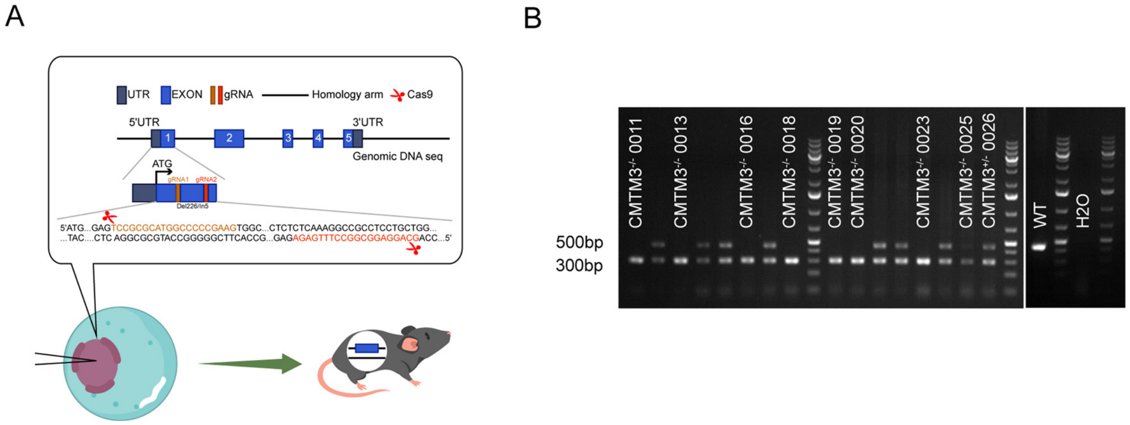

Figure EV2. Construction and identification of *Cmtm3* KO mice.

(**A**) Construction strategy of *Cmtm3* knockout mice. (**B**) Identification of *Cmtm3* homozygous, heterozygous, and wild-type mice through PCR.

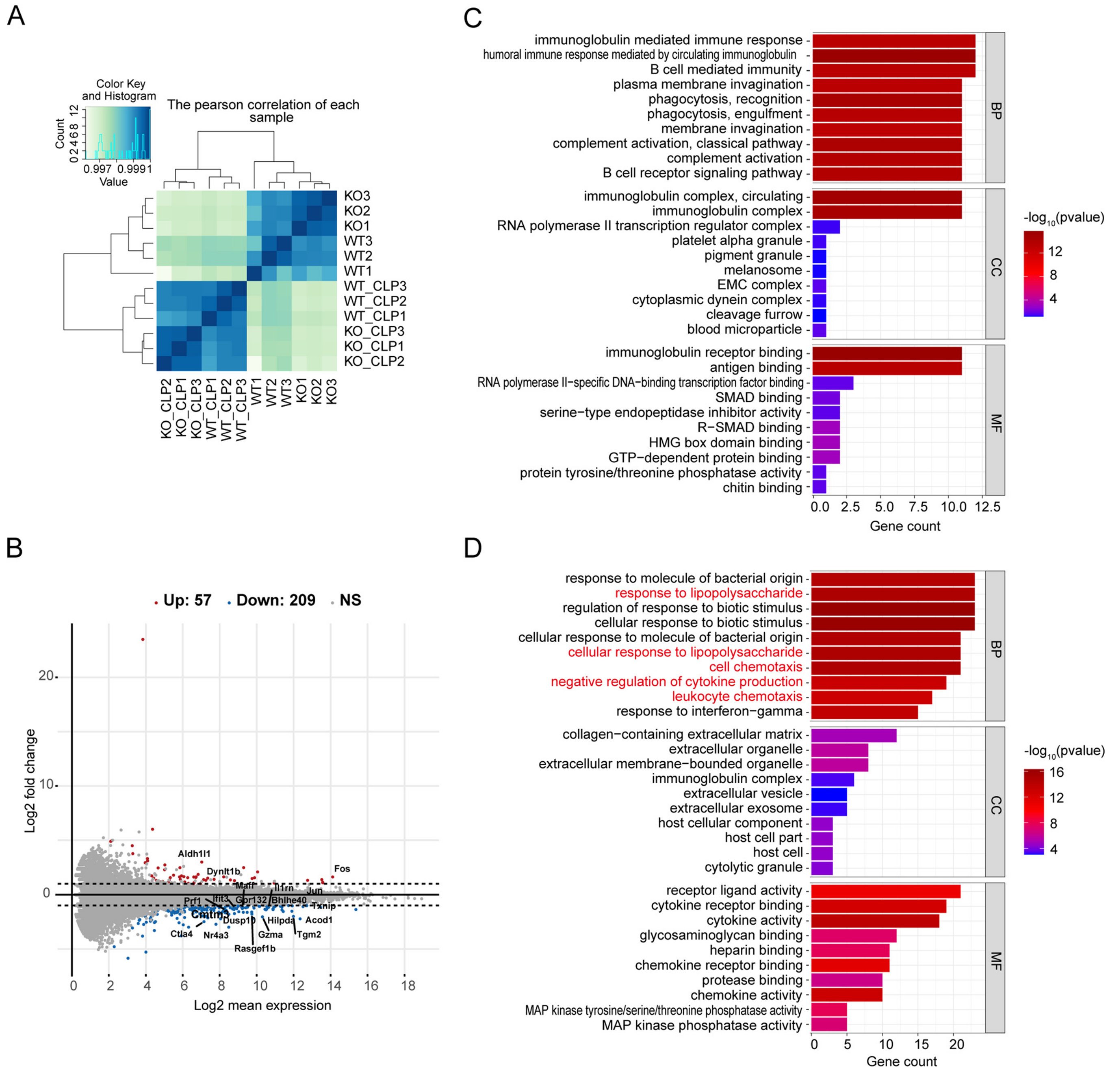

Figure EV3. RNA-seq analysis of *Cmtm3* KO mice and WT mice.

(**A**) Correlation heatmap between pairwise samples. (**B**) Volcano plot comparing differentially expressed genes between KO CLP and WT CLP ($n = 3$ mice per group, $|\log_2 FoldChange| > 1$ and P.adj < 0.05). (**C**) GO enrichment analysis of upregulated genes comparing KO CLP and WT CLP. (**D**) GO enrichment analysis of downregulated genes comparing KO CLP and WT CLP. Data information: In (**B**), data were analyzed by Student's t-est. In (**C**, **D**), data were analyzed by Hypergeometric test.

 © The Author(s)

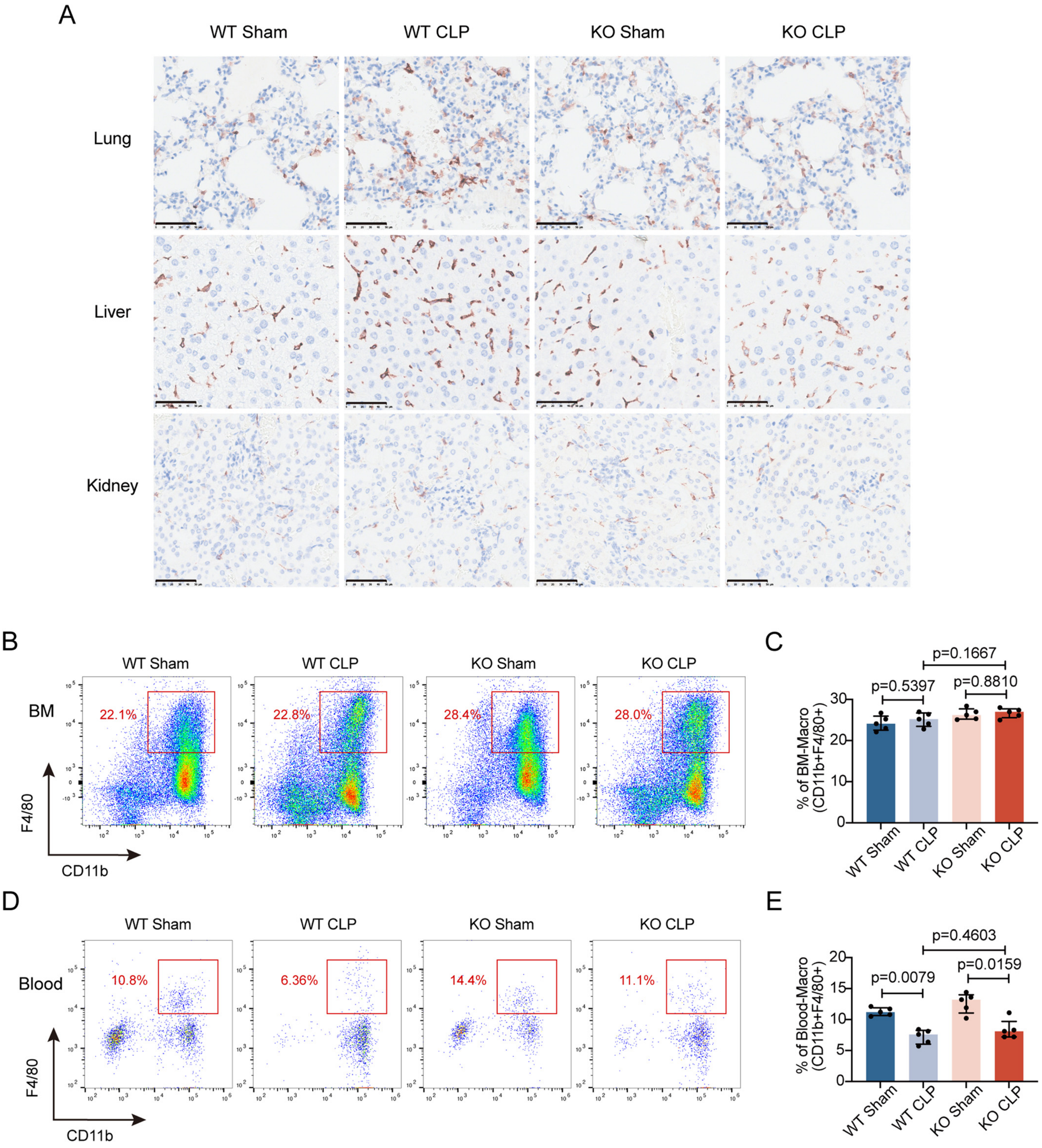

Figure EV4. The impact of *Cmtm3* KO on the distribution of macrophage populations.

(**A**) IHC staining of F4/80 of lung, liver, and kidney 24 h after Sham or CLP (scale bar: 50 μm). (**B**, **C**) BM macrophage populations in WT and KO mice 24 h after Sham or CLP ($n = 5$ mice per group). (**D**, **E**) Blood macrophage populations in WT and KO mice 24 h after Sham or CLP ($n = 5$ mice per group). Data information: In (**C**, **E**), data are expressed as the median ± IQR and were analyzed by Mann–Whitney U test.

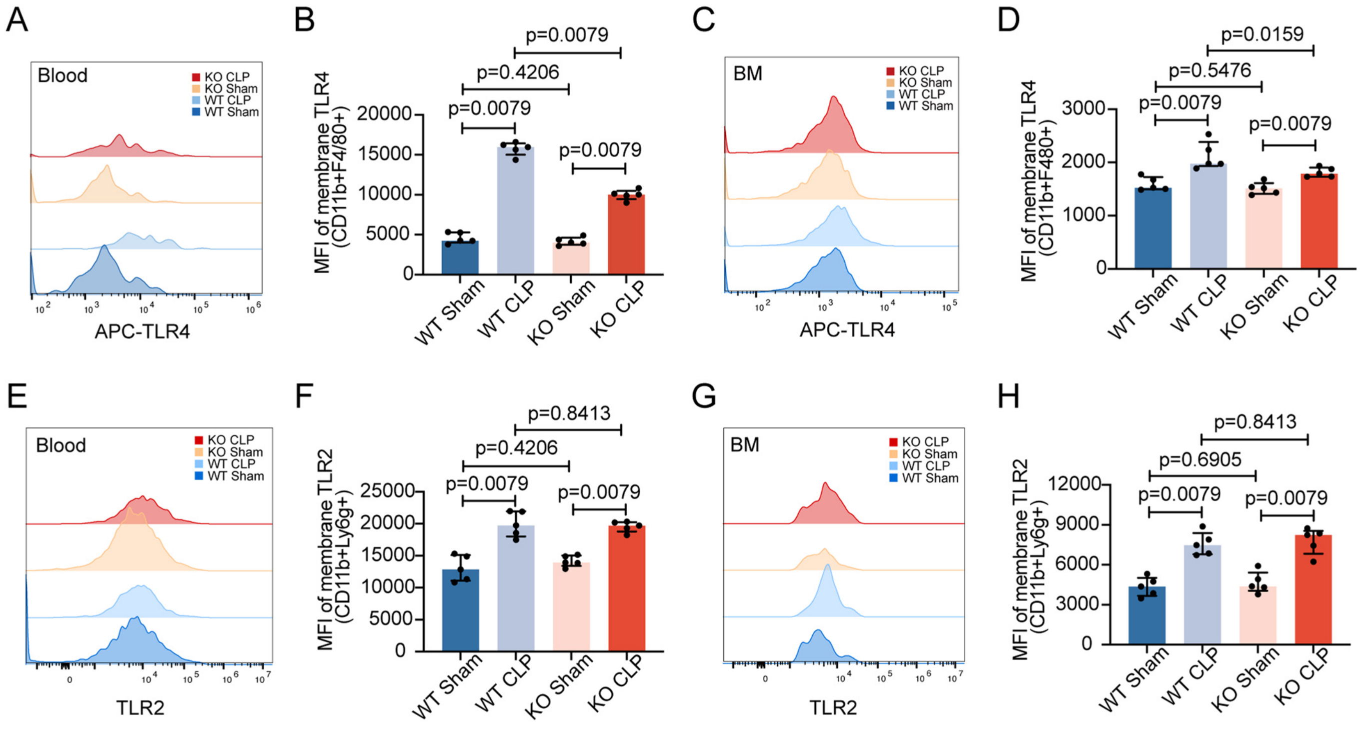

Figure EV5. The impact of *Cmtm3* KO on TLR4 expression in monocytes and TLR2 expression in neutrophils.

(**A**, **B**) TLR4 membrane expression in blood monocytes in WT and KO mice 24 h after Sham or CLP ($n = 5$ mice per group). (**C**, **D**) TLR4 membrane expression in BM monocytes in WT and KO mice 24 h after Sham or CLP ($n = 5$ mice per group). (**E**, **F**) TLR2 membrane expression in blood neutrophils in WT and KO mice 24 h after Sham or CLP ($n = 5$ mice per group). (**G**, **H**) TLR2 membrane expression in BM neutrophils in WT and KO mice 24 h after Sham or CLP ($n = 5$ mice per group). Data information: In (**B**, **D**, **F**, **H**), data are expressed as the median ± IQR and were analyzed by Mann–Whitney U test.

© The Author(s)
